# An improved gray wolf optimization algorithm solving to functional optimization and engineering design problems

**DOI:** 10.1038/s41598-024-64526-2

**Published:** 2024-06-20

**Authors:** Yihui Qiu, Xiaoxiao Yang, Shuixuan Chen

**Affiliations:** 1 School of Economics and Management, Xiamen University of Technology, Xiamen, 361024 China; 2 School of Mechanical and Automotive Engineering, Xiamen University of Technology, Xiamen, 361024 China; 3 Fujian Provincial Key Laboratory of Green Intelligent Cleaning Technology and Equipment, Xiamen, China

**Keywords:** Grey wolf algorithm, Function optimization, Engineering design optimization, Exploration and exploitation, Computational science, Software

## Abstract

As a newly proposed optimization algorithm based on the social hierarchy and hunting behavior of gray wolves, grey wolf algorithm (GWO) has gradually become a popular method for solving the optimization problems in various engineering fields. In order to further improve the convergence speed, solution accuracy, and local minima escaping ability of the traditional GWO algorithm, this work proposes a multi-strategy fusion improved gray wolf optimization (IGWO) algorithm. First, the initial population is optimized using the lens imaging reverse learning algorithm for laying the foundation for global search. Second, a nonlinear control parameter convergence strategy based on cosine variation is proposed to coordinate the global exploration and local exploitation ability of the algorithm. Finally, inspired by the tunicate swarm algorithm (TSA) and the particle swarm algorithm (PSO), a nonlinear tuning strategy for the parameters, and a correction based on the individual historical optimal positions and the global optimal positions are added in the position update equations to speed up the convergence of the algorithm. The proposed algorithm is assessed using 23 benchmark test problems, 15 CEC2014 test problems, and 2 well-known constraint engineering problems. The results show that the proposed IGWO has a balanced E&P capability in coping with global optimization as analyzed by the Wilcoxon rank sum and Friedman tests, and has a clear advantage over other state-of-the-art algorithms.

## Introduction

Swarm intelligence algorithms are a well-known class of metaheuristic algorithms that have been used to solve various engineering problems in recent years^1,2^. As compared with traditional numerical computation methods, swarm intelligence optimization algorithms have unique advantages in solving the problems associated with optimal engineering designs^3–5^. Swarm intelligence optimization algorithms have the advantages of simple principle, low requirements regarding the nature of the function, and easily computable global optimal solution. Consequently, they are widely used in solving constrained optimization problems^6–9^. For example, particle swarm optimization (PSO)^10^, whale optimization algorithm (WOA)^11^, grey wolf optimizer (GWO)^12^, tunicate swarm algorithm (TSA)^13^, seagull optimization algorithm (SOA)^14^, dingo optimization algorithm (DOA)^15^, golden jackal optimization, GJO)^16^, fox-inspired optimization algorithm (FOX)^17^, and other similar algorithms have been applied to appropriately address the functional optimization and engineering optimal design problems. However, there is room for further improvement in the quality of optimal solution, problem adaptability, and optimization stability of these algorithms. According to no-free-lunch (NFL) theorem, there is no single algorithm that can be used to effectively solve all optimization problems^18^. Therefore, exploring algorithms with higher accuracy, higher generalizability, and more stable solutions has been a focus of research community.

GWO is a meta-heuristic algorithm inspired by the hunting behavior of gray wolves^12,19^. In the iterative process of GWO, the leader wolves (α, β, and δ) guide the followers (ω) and keep narrowing down to approach the prey. During the process of searching the prey, the leader is influenced by the position of the prey, i.e., current global optimal solution, for global exploration. On the other hand, the followers perform full local exploration^20^. This act balances the mining and exploration capabilities and greatly reduces the probability of falling in local optimal solutions. Since the GWO algorithm has a simple structure, few control parameters, and high convergence accuracy^21^, more and more researchers have paid attention to the improvement and application areas of this algorithm. First, in order to achieve a good balance between exploration and exploitation, few researchers have combined GWO with other meta-heuristics. Duan et al. proposed the collaboration-based hybrid GWO-SCA optimization algorithm cHGWOSCA, and used it solve the PV model parameter extraction and 3-constraints engineering problem. The experimental results show that this algorithm has a high-performance^22^. Mohammed et al. improved the GWO algorithm by combining the advantages of whale optimization algorithm (WOA) and proposed a hybrid WOAGWO algorithm, which is used for solve an engineering problem such as pressure vessel design^23^. Mohamed et al. applied a hybrid GWO-PSO approach to address the grid-wide optimal reactive power scheduling problems. The simulation results confirm that the use of the hybrid GWO-PSO technique induces an observable improvement in the grid performance on a large scale^24^. Fatih et al. proposed three different hybrid algorithms for fusing PSO and GWO and used them for estimating the parameters of FM acoustic wave, and addressing the process flow diagram and leather nesting problems. The corresponding results verified the validity of these methods^25^.

On the other hand, few researchers have focused on strategy improvement operators to improve the mechanism of algorithms for enhancing their performance. Long et al. proposed a new stochastic dyadic-based learning strategy to help the populations jump out of the local minima. This algorithm performs well on global optimization and engineering design optimization problems^26^. Mohamed et al. proposed an improved grey wolf algorithm KMGWO based on the K-means method, which divides the population into two clusters using K-means to improve search efficiency^27^. Chen et al. introduced cat mapping and Gaussian variational perturbation strategies to effectively improve the convergence speed of GWO algorithm^28^. Gupta et al. introduced personal best history, crossover, and greedy selection strategies in their study to strengthen the global exploration capability of the GWO algorithm, and demonstrated that the resulting algorithm has a better search efficiency, solution accuracy, and convergence effect in global optimization tasks^29^. Bansal et al. used exploratory equations and inverse learning to improve the GWO algorithm. The experimental results show that this algorithm's exploration ability is better than other meta-heuristic algorithms^30^. Amir introduced two new convergence strategies to address the global optimization problem of GWO algorithm. The resulting improved algorithms, namely I-GWO and Ex-GWO, improve the performance based on the optimality seeking mechanism^31^. Wenchuan et al. introduced the inverse learning strategy and nonlinear convergence factor in standard GWO algorithm. The authors used SVM model for parameter optimization. The results show that the IGWO-SVM model has higher accuracy, efficiency, and solution stability^32^.

Due to the advancements in machine learning and intelligent optimization algorithms, more and more scholars have focused on the application of GWO to solve different real-world problems. Mohammad proposed GWO-based estimation method for photovoltaic (PV) solar cells, namely MG-GWO, which is used to extract the parameters of a single-diode photovoltaic (PV) solar cell model. The simulation results show that the MG-GWO algorithm outperforms the traditional GWO algorithm in terms of robustness and convergence speed^33^. Yan proposed a reliable and efficient emotion recognition scheme based on single channel electrocardiogram using GWO support vector machine (X-GWO-SVMs), which can be used for emotion recognition. The comparative experiments demonstrate that X-GWO-SVM algorithm is much more efficient than the existing solutions based on deep neural networks^34^. Runqian used hybrid extreme machine learning (ELM) -GWO to solve the structural problem of combined beams. It was observed that the hybrid model of GWO-ELM has a better performance as compared to separate ELM and GWO models^35^. Kun Zhang designed a distribution estimation gray wolf algorithm for low-carbon site selection path problem in the context of carbon pricing, and proved that the gray wolf algorithm with the addition of probabilistic model learning has a better optimization ability in the site selection path problem, and the constructed model can effectively reduce the total cost and carbon emissions in the context of carbon pricing^36^. Cai Yi et al. proposed a new model by combining a reverse mixed-frequency data sampling model (R-MIDAS) with GWO algorithm and used it for predicting the returns of 27 industry stock indexes and investment decisions^37^. In summary, the GWO algorithm has the ability to balance mining and exploration, accelerate the convergence speed in global search by linear convergence, improve the optimization accuracy in local search^38,39^, and avoid the emergence of local extreme points in small regions^40^. However, GWO is not very efficient for problems with more local extreme value regions and complex multibarrier problems, especially for engineering optimization problems with multiple constraints. The balance between global and local search, speed and traversability in global search, and fineness and perturbation in local search requires improvement and adjustment in accordance with different application backgrounds and types of problems. Therefore, more and more scholars have focused on new algorithms and improvement strategies to obtain a method that can be generalized for all problems.

In order to explore a better way to solve engineering optimization design problems, improve the optimization search accuracy of GWO algorithm, and expand the application space of the algorithm, this paper proposes a multi-strategy fusion of improved gray wolf optimization (GWO) algorithm. First, the initial population is optimized by using the lens imaging reverse learning algorithm, which lays the foundation for the global search. Second, a nonlinear control parameter convergence strategy based on cosine variation is proposed, which coordinates the global exploration and local development ability of the algorithm. Finally, inspired by the Tunicate Swarm Algorithm (TSA)^13^ and the particle swarm algorithm (PSO)^10^, a nonlinear tuning strategy of parameters and the personal history-based optimal position and the modified position update equation of the global optimal position to enhance the convergence speed of the algorithm. The IGWO algorithm, obtained by integrating the GWO algorithm with the proposed improvement strategy, underwent benchmark function testing and comparative experiments with CEC2014 function. It was then compared with other classical swarm intelligence optimization algorithms, as well as with algorithms improved by others to validate the effectiveness of the improvement strategy proposed in this paper. Moreover, the improved algorithm proposed in this work is used to solve the design problem of three-bar truss and vehicle side impact to further verify its engineering applicability.

The rest of the paper is organized as follows.Section "Basics and background" briefly describes the theory and major steps of conventional GWO algorithm. Section "The proposed IGWO algorithm" describes the proposed hybrid algorithm in detail. Section "Experimental environment and results" discusses the simulation results of the improved algorithm IGWO and evaluates its performance. Section "IGWO for engineering design problems" discusses the results of the improved algorithm IGWO, when used for solving 2 classical engineering design problems. Finally, section "Conclusions" summarizes the whole paper.

## Basics and background

**Figure 1:**
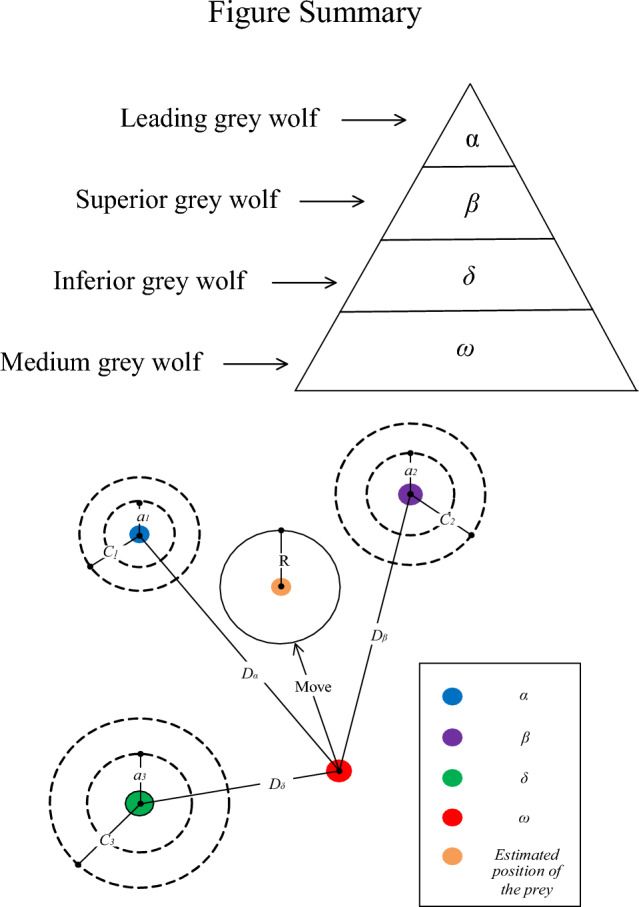
Schematic diagram of gray wolf population hierarchy and predation processes.

Gray wolves live in packs based on a rigid social hierarchy. In a pack, there is a leader known as the *α*-wolf, who is responsible for making decisions regarding hunting, food allocation, and resting places^12^. The *β*-wolves are the members of secondary level, who mainly assist the α-wolf in decision-making. The *δ*-wolves are members belonging to the tertiary level and perform scouting and sentry duties. The *ω* wolves are located at the bottom of the hierarchy and are mainly responsible for maintaining a balanced relationship within the population. The hierarchy and predation process of the gray wolf population are demonstrated in Fig. 1.

Let the dimension of the solution space of the GWO algorithm used to solve the optimization problem be *d* and the size of the gray wolf population be *N*, then, the location of the *i*-*th* gray wolf is expressed as follows:

1 $$X_{i} = \left\{ {X_{i}^{1} } \right.,X_{i}^{2} ,.....,\left. {X_{i}^{d} } \right\} ,\,i = 1,2,....,N$$

The optimal, suboptimal, and third optimal solutions in the gray wolf population are denoted as *α*, *β*, and* δ*, respectively, and the rest of the solutions are denoted as* ω*. In order to find the optimal solution or the optimal position, *ω* constantly updates the position based on the positions of *α*, *β*, and *δ*. The positions of the gray wolves are computed as:

2 $$X(t + 1) = X_{p} (t) - A \cdot |C \cdot X_{p} (t) - X(t)|$$

3 $$A = 2a \cdot r_{1} - a$$

4 $$C = 2r_{2}$$

where, *t* is the number of iterations, *X*_*p*_*(t)* is the position of the prey, *X(t)* is the position of the gray wolf after *t-*th iteration, *A* is the convergence factor, and *C* is the swing factor. *r*_*1*_ and *r*_*2*_ denote random variables between [0, 1], and *a* is the distance control parameter, whose value decreases linearly from 2 to 0 as the number of iterations increase and optimal solution is approached. This is mathematically expressed as follows:

5 $$a(t) = 2(1 - t/T_{\max } )$$

where, *T*_*max*_ denotes the maximum number of iterations.

After the prey is surrounded, the *α*-wolf leads *β*- and *δ*-wolves in its pursuit. During this process, the escape of the prey causes the position of the gray wolf population to change. Therefore, the gray wolf population uses the positions of *α*, *β*, and *δ* wolves, i.e., *X*_*α*_, *X*_*β*_, and *X*_*δ*_, respectively, to update their positions. The update process is mathematically expressed as follows:

6 $$\left\{ {\begin{array}{*{20}c} {X_{1} (t + 1) = X_{\alpha } (t) - A_{1} \cdot |C_{1} \cdot X_{\alpha } (t) - X(t)|} \\ {X_{2} (t + 1) = X_{\beta } (t) - A_{2} \cdot |C_{2} \cdot X_{\beta } (t) - X(t)|} \\ {X_{3} (t + 1) = X_{\delta } (t) - A_{3} \cdot |C_{3} \cdot X_{\delta } (t) - X(t)|} \\ \end{array} } \right.$$

7 $$X(t + 1) = \frac{{X_{1} + X_{2} + X_{3} }}{3}$$

where, *X(t* + *1)* denotes the final updated position of the gray wolf. *X*_*α*_, *X*_*β*_, and *X*_*δ*_ represent the positions of *α*, *β*, and *δ*, respectively. *X*_*1*_, *X*_*2*_, and *X*_*3*_ denote the prey positions estimated from the positions of *α*, *β*, and *δ*, respectively. Algorithm 1 presents the pseudo-code of GWO.

**Figure:**
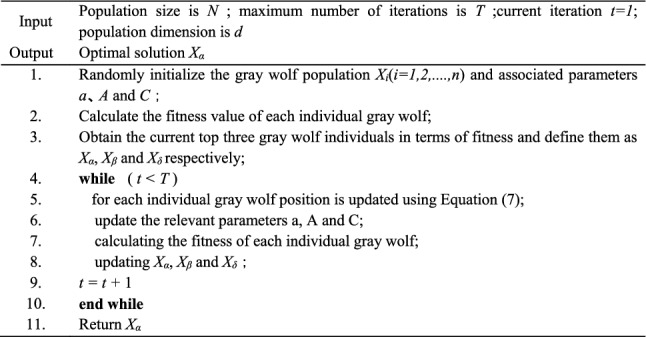
Algorithm 1: GWO Algorithm

## The proposed IGWO algorithm

In order to improve the performance of the traditional GWO algorithm, three strategies are proposed in this paper, including optimizing the initial population, modifying the nonlinear control parameters, and searching mechanism. The traditional GWO algorithm has the ability to quickly converge to the global optimum, but it easily falls into the local minima when dealing with complex optimization problems or high-dimensional problems. This performance degradation is caused due to the low exploration capability of the original GWO algorithm^30^. By optimizing the initial population, modifying the nonlinear control parameters and the search mechanism, we can enhance the exploration ability of the algorithm, thus improving its optimization ability. In summary, these strategies make the algorithm more competitive and have stronger performance.

### Lens imaging reverse learning

**Figure 2:**
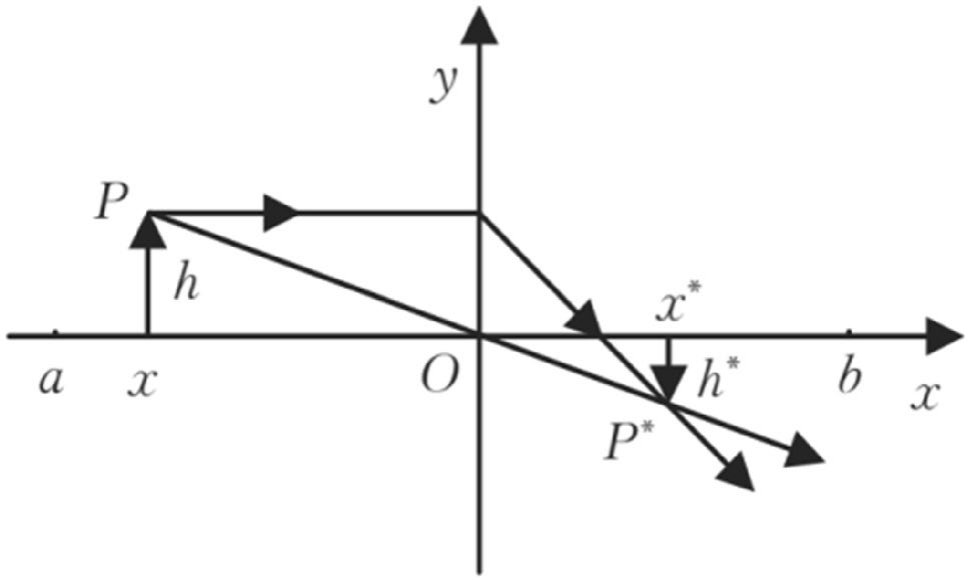
Schematic diagram of lens imagingreverse learning.

After iteration, the GWO algorithm leads to a decrease in the diversity of the population at a later stage, and the gray wolf population becomes concentrated near the position of the optimal individual. As a result, it is difficult to jump out from the local minima when the optimal individual falls into it, which leads to the phenomenon of premature termination of the algorithm, and also causes a decrease in the accuracy of the search for the optimal point^19^. In order to solve this problem, we introduce the reverse learning strategy, which considers both the solution and its opposites, and expands the search scope and seeks the optimal solution by performing a bidirectional search in the search space. Combining the reverse learning strategy with swarm intelligence algorithm effectively improves the algorithm's search^26^. The mathematical principle is shown in Fig. 2.

The individual *x* obtains its corresponding reverse point x * based on *o*, which can be obtained from the principle of lens imaging:

8 $$\frac{(a + b)/2 - x}{{x^{*} - (a + b)/2}} = \frac{h}{{h^{*} }}$$

If *k* = *h*/h *, then Eq. (8) can be rewritten as:

9 $$x^{*} = \frac{a + b}{2} + \frac{a + b}{{2k}} - \frac{x}{k}$$

Extend Eq. (9) to the *d*-dimensional optimization problem and obtain reverse learning based on lens imaging principle, as shown in Eq. (10).

10 $$x_{{}}^{d*} = \frac{{a^{d} + b^{d} }}{2} + \frac{{a^{d} + b^{d} }}{2k} - \frac{{x^{d} }}{k}$$

Therefore, in this paper, the lens imaging reverse learning strategy is used to perturb the initial population of the gray wolf population in Eq. (1) to enhance the diversity of the population and increase the probability of the algorithm jumping out of the local optimum. The mathematical expression of this strategy is:

11 $$X_{i}^{d*} = \frac{{X_{\min }^{d} + X_{\max }^{d} }}{2} + \frac{{X_{\min }^{d} + X_{\max }^{d} }}{2k} - \frac{{X_{i}^{d} }}{k}$$

where $X_{\min }^{d}$ and $X_{\max }^{d}$ denote the minimum and maximum values of the *d-*th vector in all the initial solutions, respectively, and $X_{i}^{d*}$ is the lens inverse solution of $X_{i}^{d}$. The scaling factor *k* of the lens plays a crucial role in the learning performance of lens imaging. A smaller value of *k* generates a larger range of inverse solutions, while a larger value of *k* leads to a small range of inverse solutions for localized fine search. In order to effectively utilize the characteristics of the GWO algorithm, which performs large-range exploration during early iterations and local fine search during later iterations, this work proposes a conditioning factor that varies with the number of iterations. The mathematical expression of this adjustment factor is presented below:

12 $$k(t) = \left( {1 + \left( {\frac{t}{{T_{\max } }}} \right)^{1/2} } \right)^{8}$$

where, *t* is the current number of iterations and *T*_*max*_ is the maximum number of iterations. *k* plays a role in regulating the inverse solution. As the number of iterations increases, the value of *k* becomes larger, resulting in a smaller range of inverse solutions for lens imaging inverse learning. This conditioning mechanism enhances the fine search for localized locations during later iterations of the algorithm.

### Modified control parameter

**Figure 3:**
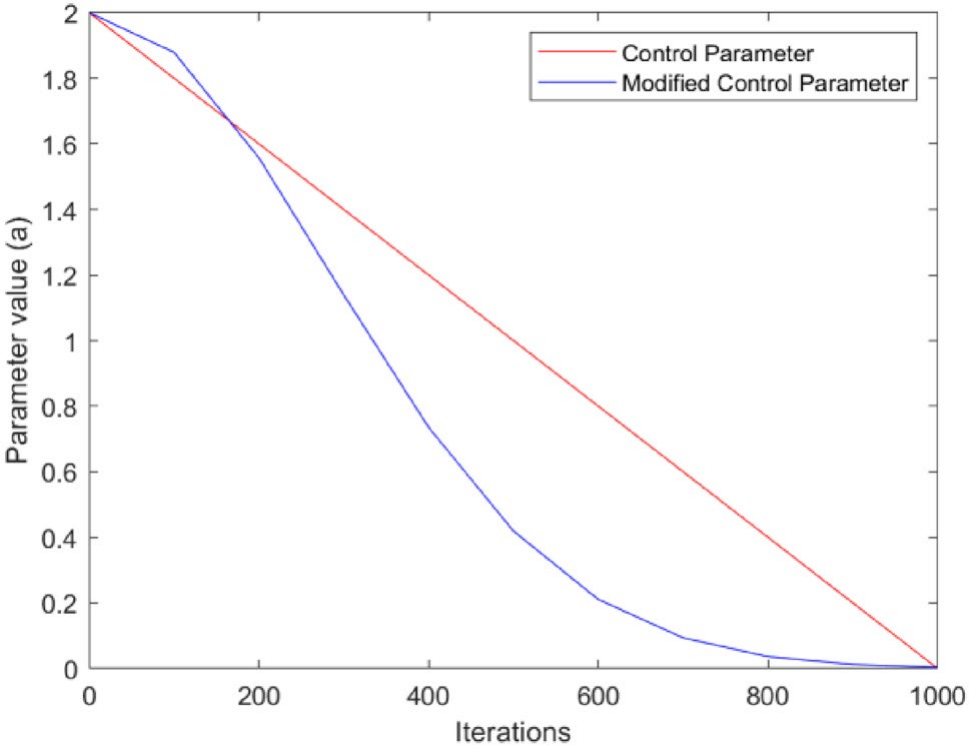
Linearly and non-linearly decreasing curve for parameter *a.*

When |A|≥ 1, the gray wolves in the GWO algorithm expand the search range in order to perform a global search. On the other hand, when |A|< 1, the individuals in the gray wolf population narrow their search range to finely search for the optimal solution. From (5), it is evident that the distance control parameter *a* determines the value of the variable A. The value of the parameter *a* plays a key role in assisting the algorithm to perform global and local search. However, the parameter *a* decreases from 2 to 0 in a linear manner as the number of iterations increases. This linear change strategy does not accurately reflect the actual nonlinear search process, which may cause the algorithm to fall into a local optimum^38,39^. In response to this deficiency, this paper proposes a nonlinear control parameter convergence strategy. This is mathematically expressed as follows:

13 $$a = (a_{initial} - a_{end} )*\exp \left( { - \frac{{t^{2} }}{{(K*T_{\max } )^{2} }}} \right) + a_{end}$$

where, *t* denotes the current number of iterations, *T*_*max*_ denotes the maximum number of iterations, *K* is the nonlinear modulation index, and *a*_*initial*_ and *a*_*end*_ are the initial and final values of parameter a, respectively. In order to achieve better exploitation goals, in this work, we fix the value of k to 0.3 and set the values of *a*_*initial*_ and *a*_*end*_ to 2 and 0, respectively. As shown in Fig. 3, this paper compares the linear and nonlinear control parameters. It can be observed in Fig. 3 that the linear behavior of the control parameters indicates 50% for global exploration and 50% for local mining, while the nonlinear behavior of the control parameters suggests 25% for global exploration and 75% for local mining. Therefore, the use of nonlinear control parameters focuses more on mining, which improves the local search and reduces the possibility of skipping the true solution.

### Modified search mechanism

The information provided by *α*, *β*, and *δ* wolves are important factors in updating the position of *ω* wolves. In the traditional GWO algorithm, the average value of the estimated positions of the three best search agents (*α*, *β* and *δ*) is used to obtain the new position of the wolf^20^. Logically, the traditional GWO provides a new position at the center of mass of the convex region surrounded by the orientation points obtained by *α*, *β*, and *δ* wolves. Therefore, the new position of the wolf is oriented in the same direction as the estimated positions of the *α*, *β*, and *δ* wolves. However, this logic does not work effectively when the lead wolves are stuck in a local minima or are far from the optimum^29^. Therefore, inspired by the TSA^13^ and PSO^10^ algorithms, a nonlinear adjustment strategy for the parameters and a modified position update equation based on the individual's historical optimal position and the global optimal position are added to reduce the possibility of falling into a local optimum. Among them, the position update equation of TSA algorithm is shown in (14), and the position update equation of PSO algorithm is shown in (15).

14 $$X(t + 1) = \frac{X(t) + X(t + 1)}{{2 + rand()}}$$

15 $$X_{i} = X_{i} + v_{i}$$

where $v_{i} = v_{i} + c_{3} *rand()*(pbest_{i} - x_{i} ) + c_{4} *rand()*(gbest_{i} - x_{i} )$, *v*_*i*_ is the particle velocity, *rand()* is a random number between (0, 1), and *c*_*1*_ and *c*_*2*_ are learning factors. Usually *c*_*1*_ = *c*_*2*_ = *2*. *pbest*_*i*_*(t)* denotes the individual historical best position and *gbest*_*i*_*(t)* denotes the global historical best position.

In summary, this paper proposes a new search mechanism expressed as:

16 $$X(t + 1) = (w_{1} *X_{1} + w_{2} *X_{2} + \left( {b_{1} *r_{3} *(pbest_{i} (t) - X(t) + b_{2} *r_{4} *(X_{1} - X(t)} \right)/(randn() + 2)$$

where *X*_*1*_ and *X*_*2*_ denote the prey positions estimated from the positions of *α* and *β*, respectively. *b*_*1*_ and *b*_*2*_ denote the individual memory coefficient and group communication coefficient, respectively. *b*_*1*_ = *b*_*2*_ = 0.5. *r*_*3*_ = *r*_*4*_ are uniformly distributed random numbers between [0, 1], *pbest*_*i*_*(t)* denotes the best position in the history of the individual, and *randn()* denotes the random perturbation term with a normal distribution. *w*_*1*_ and *w*_*2*_ are the inertia weights, which are expressed as follows:

17 $$\begin{array}{*{20}c} {w_{1} = \frac{{f_{\alpha } }}{{f_{\alpha } + f_{\beta } }}} & {w_{2} = \frac{{f_{\beta } }}{{f_{\alpha } + f_{\beta } }}} \\ \end{array}$$

where *f*_*α*_ and *f*_*β*_ denote the adaptation values of *α*-wolf and *β*-wolf during *t-*th iteration, respectively.

By embedding all the aforementioned strategies in the original GWO algorithm, a new variant of GWO called IGWO is proposed. Algorithm 2 presents the pseudo-code of IGWO containing all the strategies.

**Figure:**
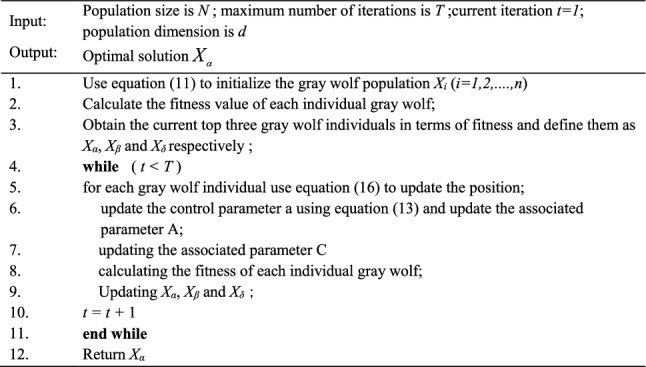
Algorithm 2: IGWO algorithm

## Experimental environment and results

In this work, 23 optimization benchmark problems with different characteristics^12,41^, 15 optimization problems from CEC2014^30,42^, and two engineering problems are used to evaluate the performance of the proposed IGWO algorithm. Table 1 presents benchmark problems, including 7 single-peak problems (*F*_*1*_*-F*_*7*_), 6 multi-peak problems (*F*_*8*_*-F*_*13*_), and 10 multi-peak problems with fixed dimensions (*F*_*14*_*-F*_*23*_). As presented in Table 2, 15 functions in CEC2014 are selected in this paper, including 1 single-peak function (*F*_*03*_), 7 multi-peak functions (*F*_*06*_, *F*_*08*_*-F*_*11*_, *F*_*13*_, *F*_*16*_), 1 hybrid function (*F*_*19*_), and 6 combined functions (*F*_*23*_*-F*_*24*_, *F*_*26*_*-F*_*29*_). Where Dimension (*N*) denotes the number of decision variables and Range (*D*) denotes the boundary of decision variables. The optimal values corresponding to each test function are given in Tables 1 and 2. The single-peak test function has only one global optimum, which is useful for testing the local search ability of the algorithm. Unlike the single-peak problem, the multi-peak problem has multiple local optima. These local optima increase with an increase in the number of dimensions. The global search ability of the proposed algorithm can be tested by solving the multi-peak problem. In contrast, multi-peak problems with fixed dimensions, combined functions, and hybrid functions are used to demonstrate the exploration and exploitation capabilities of the proposed method.

**Table 1:** Benchmark problems.

Function		Range	Dimension	Optimal value
F1	$F_{1} = \sum\nolimits_{i = 1}^{n} {x_{i}^{2} }$	[− 100, 100]	30/100/500/1000	0
F2	$F_{2} = \sum\nolimits_{i = 1}^{n} {|x_{i} | + \prod\nolimits_{i = 1}^{n} {|x_{i} |} }$	[− 10, 10]	30/100/500/1000	0
F3	$F_{3} = \sum\nolimits_{i = 1}^{n} {(\sum\nolimits_{j = 1}^{i} {x_{j} )^{2} } }$	[− 100, 100]	30/100/500/1000	0
F4	$$F_{4} = \max_{i} \{ \begin{array}{*{20}c} {|x_{i} |,} & {1 \le i \le n\} } \\ \end{array}$$	[− 100, 100]	30/100/500/1000	0
F5	$F_{5} = \sum\nolimits_{i = 1}^{n - 1} {[100(x_{i + 1} - x_{i}^{2} )^{2} + (x_{i} - 1)^{2} ]}$	[− 30, 30]	30/100/500/1000	0
F6	$F_{6} = \sum\nolimits_{i = 1}^{n} {([x_{i} + 0.5])^{2} }$	[− 100, 100]	30/100/500/1000	0
F7	$F_{7} = \sum\nolimits_{i = 1}^{n} {ix_{i}^{4} + random[0,1)}$	[− 1.28, 1.28]	30/100/500/1000	0
F8	$F_{8} = \sum\nolimits_{i = 1}^{n} { - x_{i} \sin (\sqrt {|x_{i} |} }$	[− 500, 500]	30/100/500/1000	− 418.9829 × D
F9	$F_{9} = \sum\nolimits_{i = 1}^{n} {[x_{i}^{2} - 10\cos (2\pi x_{i} ) + 10]}$	[− 5.12, 5.12]	30/100/500/1000	0
F10	$F_{10} = - 20\exp \left( { - 0.2\sqrt {\frac{1}{n}\sum\nolimits_{i = 1}^{n} {x_{i}^{2} } } } \right) - \exp \left( {\frac{1}{n}\sum\nolimits_{i = 1}^{n} {\cos (2\pi x_{i} )} } \right) + 20 + e$	[− 32, 32]	30/100/500/1000	0
F11	$F_{11} = \frac{1}{4000}\sum\nolimits_{i = 1}^{n} {x_{i}^{2} - \prod\nolimits_{i = 1}^{n} {\cos \left( {\frac{{x_{i} }}{\sqrt i }} \right)} } + 1$	[− 600, 600]	30/100/500/1000	0
F12	$$\begin{gathered} F_{12} = \frac{\pi }{n}\{ 10\sin^{2} (\pi y_{i} ) \hfill \\ \begin{array}{*{20}c} {} & {} \\ \end{array} + \sum\nolimits_{i = 1}^{n - 1} {(y_{i} - 1)^{2} } [1 + 10\sin^{2} (\pi y_{i + 1} )] \hfill \\ \begin{array}{*{20}c} {} & {} \\ \end{array} + (y_{n} - 1)^{2} ] + \sum\nolimits_{i = 1}^{n} {(ux_{i} ,10,100,4)} , \hfill \\ y_{i} = 1 + \frac{1}{4}(x_{i} + 1) \hfill \\ u(x_{i} ,a,k,m) = \left\{ {\begin{array}{*{20}c} {k(x_{i} - a)^{m} ,\begin{array}{*{20}c} {} & {x_{i} > a,} \\ \end{array} } \\ {0,\begin{array}{*{20}c} {} & {} \\ \end{array} - a \le x_{i} \le a,} \\ {k( - x_{i} - a)^{m} ,x_{i} < - a.} \\ \end{array} } \right. \hfill \\ \end{gathered}$$	[− 50, 50]	30/100/500/1000	0
F13	$$\begin{gathered} F_{13} = 0.1\{ \sin^{2} (3\pi x_{1} ) \hfill \\ \begin{array}{*{20}c} {} & {} \\ \end{array} + \sum\nolimits_{i = 1}^{n - 1} {(x_{i} - 1)^{2} [1 + \sin^{2} (3\pi x_{i + 1} )]} \hfill \\ \begin{array}{*{20}c} {} & {} \\ \end{array} + (x_{n} - 1)[1 + \sin^{2} (2\pi x_{n} )]\} \hfill \\ \begin{array}{*{20}c} {} & {} \\ \end{array} + \sum\nolimits_{i = 1}^{n} {u(x_{i} ,5,100,4)} \hfill \\ \end{gathered}$$	[− 50, 50]	30/100/500/1000	0
F14	$F_{14} = \left[ {\frac{1}{500} + \sum\nolimits_{j = 1}^{25} {\frac{1}{{j + \sum\nolimits_{i = 1}^{2} {(x_{i} - a_{ij} )^{6} } }}} } \right]^{ - 1}$	[− 65.536, 65.536]	2	1
F15	$F_{15} = \sum\nolimits_{i = 1}^{11} {\left[ {a_{i} - \frac{{x_{1} (b_{i}^{2} + b_{i} x_{2} )}}{{b_{i}^{2} + b_{i} x_{3} + x_{4} }}} \right]}^{2}$	[− 5, 5]	4	0.0003075
F16	$F_{16} = 4x_{1}^{2} - 2.1x_{1}^{4} + \frac{1}{3}x_{1}^{6} + x_{1} x_{2} - 4x_{2}^{2} + 4x_{2}^{4}$	[− 5, 5]	2	− 1.0316285
F17	$F_{17} = \left( {x_{2} - \frac{5.1}{{4\pi^{2} }}x_{1}^{2} + \frac{5}{\pi }x_{1} - 6} \right)^{2} + 10\left( {1 - \frac{1}{8\pi }} \right)\cos x_{1} + 10$	[− 5,10] × [0,15]	2	0.398
F18	$$\begin{gathered} F_{18} = [1 + (x_{1} + x_{2} + 1)^{2} (19 - 14x_{1} + 3x_{1}^{2} - 14x_{2} \hfill \\ \begin{array}{*{20}c} {} & { + 6x_{1} x_{2} + 3x_{2}^{2} )] \times [30 + (2x_{1} - 3x_{2} )^{2} (18} \\ \end{array} - 32x_{1} \hfill \\ \begin{array}{*{20}c} {} & { + 12x_{1}^{2} + 48x_{2} - 36x_{1} x_{2} + 27x_{2}^{2} )]} \\ \end{array} \hfill \\ \end{gathered}$$	[− 2, 2]	2	3
F19	$F_{19} = - \sum\nolimits_{i = 1}^{4} {c_{i} \exp \left( { - \sum\nolimits_{j = 1}^{3} {a_{ij} (x_{j} - p_{ij} )^{2} } } \right)}$	[0, 1]	4	− 3.86
F20	$F_{20} = - \sum\nolimits_{i = 1}^{4} {c_{i} \exp \left( { - \sum\nolimits_{j = 1}^{6} {a_{ij} (x_{j} - p_{ij} )^{2} } } \right)}$	[0, 1]	6	− 3.32
F21	$F_{21} = - \sum\nolimits_{{}}^{{}} {[(X - a_{i} )(} X - a_{i} )^{T} + c_{i} ]^{ - 1}$	[0, 10]	4	− 10.1532
F22	$F_{22} = - \sum\nolimits_{i = 1}^{7} {[(X - a_{i} )(X - a_{i} ){}^{T} + c_{i} ]^{ - 1} }$	[0, 10]	4	− 10.4028
F23	$F_{23} = - \sum\nolimits_{i = 1}^{10} {[(X - a_{i} )} (X - a_{i} )^{T} + c_{i} ]^{ - 1}$	[0, 10]	4	− 10.5363

**Table 2:** CEC2014 problem.

Function	Function name	Optimal value	Type
F03	Rotated discus function	300	Single-peak function
F06	Shifted and rotated weierstrass function	600	Multi-peak function
F08	Shifted Rastrigin’s function	800	
F09	Shifted and rotated Rastrigin’s function	900	
F10	Shifted Schwefel’s function	1000	
F11	Shifted and rotated Schwefel’s function	1100	
F13	Shifted and rotated Happycat function	1300	
F16	Shifted and rotated expanded Scaffer’s F6 function	1600	
F19	Hybrid Function 3 (N = 4)	1900	Combinatorial functions
F23	Composition Function 1 (N = 5)	2300	Hybrid functions
F24	Composition Function 2 (N = 3)	2400	
F26	Composition Function 4 (N = 5)	2600	
F27	Composition Function 5 (N = 5)	2700	
F28	Composition Function 6 (N = 5)	2800	
F29	Composition Function 7 (N = 3)	2900	

The experiments performed in this work are divided into 4 parts. First, the proposed IGWO is compared with other swarm intelligence optimization algorithms and other improved GWO algorithms based on a 30-dimensional benchmark test problem.In the second part, the proposed IGWO is compared with other swarm intelligence optimization algorithms and other improved GWO algorithms based on 100, 500, and 1000 dimensional scalable test problems of* F*_*1*_*-F*_*13*_. In the third part, the proposed IGWO algorithm is used to solve the CEC2014 problem. Finally, the proposed IGWO algorithm is applied to 2 classical engineering problems.

All these experiments are performed in the same environment (parameter settings). The parameter settings of all meta-heuristic optimization algorithms are set based on the literature, as shown in Table 3. The maximum number of iterations *T*_*max*_ is set to 1000 for all functions, the population size is set to 30 individuals, and the population size is initialized in the same manner for all algorithms being compared in this work. All the results obtained for the benchmark problem, the CEC2014 problem, and the engineering problem are averaged over 30 epochs. The experimental results are analyzed based on two criteria: (1) the mean and standard deviation (Std) values of the best results obtained during the 30 trials of the algorithms, and (2) statistical analysis based on Wilcoxon rank sum and Friedman's tests. In addition, all the experiments are performed on the same machine with an i5 processor with 16 GB of RAM and Win10 64-bit operating system. The experiments are implemented in MATLAB.

**Table 3:** Parameter setting of the various algorithms.

S. No.	Algorithms	Parameter setting
1	GWO	a = [0, 2]
2	MFO^43^	b = 1
3	SHO^44^	E = [0, 1]; M = [0.5, 1]
4	TSA^13^	P_max_ = 4; P_min_ = 1
5	PSO^10^	c_1_ = c_2_ = 2
6	DOA^15^	P = 0.5; Q = 0.7; β_1_ = [-2,2]
7	GJO^16^	C_1_ = 1.5; E_1_ = [0, 1.5]
8	SOA^17^	*f*_*c*_ = [0, 2]; u = 1; v = 0.1
9	WOA^11^	b = 1; *p* = [0, 1]; P = 0.5
10	catGWO^28^	User defined parameters selected from the original papers
11	CPSOGSA^45^	

### Analysis of different strategies

In this subsection, to verify the impact of each strategy in the IGWO, three different combinations are added in the GWO algorithm such as Strategy-1, Strategy-2, and Strategy-3. In Strategy-1, only lens imaging reverse learning is added to the GWO algorithm. In Strategy-2, only a modified control parameter is added to the GWO algorithm. In Strategy-3, only a new location search mechanism is added to the GWO algorithm. IGWO means that all strategies are added to the GWO algorithm. Same benchmark problems (*F*_*1*_-*F*_*23*_) have been used to verify the impact of each strategy. The parameter settings are as same as in section "Experimental environment and results", and each benchmark problem runs 30 times, individually. The specific experimental results are shown in Table 4.

**Table 4:** Obtained results by Strategy-1, Strategy-2, Strategy-3, and IGWO well-known benchmark problems.

	Fun	IGWO	GWO	Strategy-1	Strategy-2	Strategy-3
F_1_	Mean	**0.00E+00**	2.19E−58	6.45E−59	5.08E−87	**0.00E+00**
	Std	**0.00E+00**	1.03E−57	2.08E−58	1.59E−86	**0.00E+00**
F_2_	Mean	**0.00E+00**	1.09E−34	8.25E−35	1.73E−50	**0.00E+00**
	Std	**0.00E+00**	9.99E−35	8.22E−35	3.07E−50	**0.00E+00**
F_3_	Mean	**0.00E+00**	1.48E−13	1.32E−14	2.78E−15	**0.00E+00**
	Std	**0.00E+00**	7.90E−13	4.62E−14	1.53E−14	**0.00E+00**
F_4_	Mean	**0.00E+00**	2.31E−14	1.15E−14	7.17E−24	**0.00E+00**
	Std	**0.00E+00**	4.60E−14	1.79E−14	1.43E−23	**0.00E+00**
F_5_	Mean	**9.58E−01**	2.65E+01	2.65E+01	2.61E+01	4.78E+01
	Std	**1.08E−01**	8.86E−01	5.89E−01	5.34E−01	5.24E−01
F_6_	Mean	**7.81E−08**	6.40E−01	5.38E−01	1.23E−01	1.26E−06
	Std	**8.44E−08**	3.72E−01	3.51E−01	2.88E−01	1.96E−06
F_7_	Mean	**3.08E−05**	8.03E−04	7.06E−04	6.25E−04	3.67E−05
	Std	**2.42E−05**	4.99E−04	3.45E−04	3.90E−04	2.96E−05
F_8_	Mean	**-1.26E+04**	-5.83E+03	-5.80E+03	-3.59E+03	-1.25E+04
	Std	**3.28E−03**	1.02E+03	8.09E+02	3.39E+02	1.05E−02
F_9_	Mean	**0.00E+00**	5.15E−01	3.55E−01	**0.00E+00**	**0.00E+00**
	Std	**0.00E+00**	1.58E+00	1.36E+00	**0.00E+00**	**0.00E+00**
F_10_	Mean	**8.88E−16**	1.59E−14	1.49E−14	7.52E−15	**8.88E−16**
	Std	**0.00E+00**	2.41E−15	2.38E−15	1.23E−15	**0.00E+00**
F_11_	Mean	**0.00E+00**	4.67E−03	2.82E−03	**0.00E+00**	**0.00E+00**
	Std	**0.00E+00**	1.08E−02	6.57E−03	**0.00E+00**	**0.00E+00**
F_12_	Mean	**3.96E−09**	3.50E−02	3.31E−02	4.32E−02	2.07E−07
	Std	**6.16E−09**	2.15E−02	1.83E−02	2.26E−02	2.97E−07
F_13_	Mean	**8.99E−08**	5.05E−01	5.04E−01	4.93E−01	2.28E−06
	Std	**1.26E−07**	1.80E−01	1.95E−01	1.78E−01	3.46E−06
F_14_	Mean	**1.78E+00**	3.86E+00	4.61E+00	4.84E+00	5.27E+00
	Std	**2.96E+00**	4.43E+00	4.76E+00	4.61E+00	5.72E+00
F_15_	Mean	**3.55E−04**	5.78E−03	5.56E−03	2.49E−03	1.67E−03
	Std	**8.30E−10**	8.95E−03	8.01E−03	6.06E−03	2.49E−09
F_16_	Mean	**-1.03E+00**	**-1.03E+00**	**-1.03E+00**	**-1.03E+00**	**-1.03E+00**
	Std	**6.78E−16**	8.35E−09	5.37E−09	2.72E−09	4.52E−09
F_17_	Mean	**3.98E−01**	**3.98E−01**	**3.98E−01**	**3.98E−01**	**3.98E−01**
	Std	**0.00E+00**	6.58E−07	1.47E−07	3.57E−07	1.29E−07
F_18_	Mean	**3.00E+00**	**3.00E+00**	**3.00E+00**	**3.00E+00**	**3.00E+00**
	Std	**1.05E−06**	1.10E−05	1.50E−05	2.55E−06	1.07E−05
F_19_	Mean	**-3.86E+00**	**-3.86E+00**	**-3.86E+00**	**-3.86E+00**	**-3.86E+00**
	Std	**2.70E−15**	1.95E−03	2.44E−03	1.18E−03	2.33E−03
F_20_	Mean	**-3.29E+00**	-3.27E+00	-3.26E+00	-3.27E+00	-3.28E+00
	Std	**5.54E−02**	6.17E−02	6.50E−02	6.70E−02	5.90E−02
F_21_	Mean	**− 10.1532**	-9.31E+00	-9.13E+00	-9.33E+00	-9.64E+00
	Std	**4.01E−05**	1.92E+00	1.93E+00	1.39E+00	1.54E+00
F_22_	Mean	**-10.4028**	-10.4025	-10.4025	-10.2267	-10.0482
	Std	**3.71E−05**	1.34E+00	2.00E−04	3.52E−04	2.70E−04
F_23_	Mean	**-10.5363**	-1.03E+01	-1.05E+01	-1.04E+01	-1.03E+01
	Std	**3.30E−05**	1.48E+00	3.62E−04	9.87E−01	1.48E+00

Significant are in value [bold].

The experimental results from Table 4 demonstrate that the proposed IGWO outperforms the other three strategies on 23 benchmark problems and is capable of achieving near-optimal values on multiple problems. Strategy-3 is considered a suboptimal optimizer, yet all three strategies perform better than the original GWO algorithm. Strategy-1 introduces a lens imaging reverse learning strategy to enhance population coverage and uniformity, but the performance improvement is not significant. Strategy-2 incorporates a nonlinear convergence strategy, significantly enhancing the search ability and efficiency during the exploration process, leading to significant improvements in average fitness values and standard deviations across multiple problems. Strategy-3 introduces a new position update strategy, significantly boosting the algorithm's capability to avoid local optima, though it remains a suboptimal optimizer. The IGWO algorithm combines the advantages of these three strategies and leverages optimization performance to the highest level, demonstrating outstanding performance in function optimization.

### Comparison using benchmark functions

In this subsection, the performance of IGWO is analyzed and compared with other swarm intelligence optimization algorithms based on the mean and standard deviation (Std) of 30 independent epochs. The best results are highlighted in bold. Table 5 presents the optimization search results obtained using the proposed IGWO, GWO^12^, MFO^43^, SHO^44^, TSA^13^, PSO^10^, DOA^15^, GJO^16^, SOA^17^, WOA^11^, FOX^17^, catGWO^28^, and CPSOGSA^45^ for 23 benchmark functions.

**Table 5:** Fitness results of different algorithms on 23 benchmark functions.

	Fun	IGWO	GWO	MFO	SHO	TSA	PSO	DOA	GJO	SOA	WOA	FOX	Cat GWO	CPSO GSA
F_1_	Mean	**0.00E+00**	2.19E−58	2.00E+03	1.66E+03	1.55E−47	2.83E−01	4.43E−117	1.95E−112	9.53E−28	3.11E−147	**0.00E+00**	**0.00E+00**	1.15E−18
	Std	**0.00E+00**	1.03E−57	4.06E+03	4.61E+03	3.81E−47	1.89E−01	1.81E−116	8.18E−112	1.91E−27	1.70E−146	**0.00E+00**	**0.00E+00**	1.79E−19
F_2_	Mean	**0.00E+00**	1.09E−34	2.60E+01	3.23E+01	1.27E−28	1.02E+00	5.66E−88	1.95E−66	1.10E−17	6.78E−103	**0.00E+00**	**0.00E+00**	3.61E+01
	Std	**0.00E+00**	9.99E−35	2.47E+01	2.01E+01	4.19E−28	3.92E−01	3.10E−87	2.26E−66	1.64E−17	3.58E−102	**0.00E+00**	**0.00E+00**	4.96E+01
F_3_	Mean	**0.00E+00**	1.48E−13	1.71E+04	1.61E+04	1.15E−10	8.67E+01	1.42E−116	2.07E−39	2.50E−14	1.98E+04	**0.00E+00**	**0.00E+00**	1.27E+03
	Std	**0.00E+00**	7.90E−13	1.08E+04	1.04E+04	3.84E−10	2.78E+01	7.77E−116	8.49E−39	9.94E−14	1.09E+04	**0.00E+00**	**0.00E+00**	7.91E+02
F_4_	Mean	**0.00E+00**	2.31E−14	6.55E+01	6.65E+01	4.92E−03	1.57E+00	2.10E−84	2.27E−33	7.06E−08	4.36E+01	**0.00E+00**	**0.00E+00**	2.97E+01
	Std	**0.00E+00**	4.60E−14	1.24E+01	8.53E+00	8.94E−03	2.17E−01	1.15E−83	4.61E−33	3.17E−07	2.92E+01	**0.00E+00**	**0.00E+00**	9.59E+00
F_5_	Mean	**9.58E−01**	2.65E+01	9.86E+03	1.83E+04	2.81E+01	2.62E+02	2.89E+01	2.73E+01	2.81E+01	2.71E+01	2.97E+01	2.87E+01	3.52E+01
	Std	**1.08E−01**	8.86E−01	2.72E+04	3.62E+04	1.04E+00	1.01E+02	4.26E−02	7.54E−01	6.20E−01	6.41E−01	3.85E−02	2.84E−01	2.16E+01
F_6_	Mean	**7.81E−08**	6.40E−01	2.99E+03	2.01E+03	3.73E+00	2.80E−01	5.69E+00	2.54E+00	3.20E+00	9.07E−02	2.83E−03	5.17E+00	3.30E+02
	Std	**8.44E−08**	3.72E−01	5.34E+03	4.87E+03	6.01E−01	1.76E−01	8.89E−01	3.13E−01	4.83E−01	9.88E−02	1.12E−03	4.00E−01	1.81E+03
F_7_	Mean	**3.08E−05**	8.03E−04	4.20E+00	7.24E+00	4.36E−03	3.29E+00	1.36E−04	1.97E−04	1.33E−03	1.36E−03	9.09E−05	4.63E−05	7.09E−02
	Std	**2.42E−05**	4.99E−04	7.37E+00	1.63E+01	1.76E−03	3.63E+00	1.83E−04	1.85E−04	9.26E−04	1.62E−03	7.42E−05	4.33E−05	2.34E−02
F_8_	Mean	**-1.26E+04**	-5.83E+03	-8.45E+03	-8.81E+03	-6.20E+03	-6.89E+03	-4.94E+03	-4.31E+03	-5.34E+03	-1.10E+04	-7.14E+03	-6.07E+03	-6.44E+03
	Std	**3.28E−03**	1.02E+03	1.01E+03	7.97E+02	6.16E+02	1.07E+03	8.85E+02	1.18E+03	7.48E+02	1.81E+03	6.64E+02	1.59E+03	5.87E+02
F_9_	Mean	**0.00E+00**	5.15E−01	1.62E+02	1.59E+02	1.63E+02	1.15E+02	**0.00E+00**	**0.00E+00**	6.22E−01	**0.00E+00**	**0.00E+00**	**0.00E+00**	1.43E+02
	Std	**0.00E+00**	1.58E+00	2.85E+01	4.38E+01	3.77E+01	3.16E+01	**0.00E+00**	**0.00E+00**	2.11E+00	**0.00E+00**	**0.00E+00**	**0.00E+00**	2.76E+01
F_10_	Mean	**8.88E−16**	1.59E−14	1.63E+01	1.54E+01	1.66E+00	1.11E+00	**8.88E−16**	4.56E−15	2.00E+01	4.32E−15	1.59E−14	**8.88E−16**	1.40E+00
	Std	**0.00E+00**	2.41E−15	6.67E+00	6.49E+00	1.60E+00	5.12E−01	**0.00E+00**	6.49E−16	1.77E−03	2.38E−15	3.45E−15	**0.00E+00**	4.20E+00
F_11_	Mean	**0.00E+00**	4.67E−03	1.81E+01	3.92E+01	6.14E−03	2.25E−02	**0.00E+00**	**0.00E+00**	4.03E−03	**0.00E+00**	**0.00E+00**	**0.00E+00**	4.07E+00
	Std	**0.00E+00**	1.08E−02	3.67E+01	6.13E+01	7.08E−03	1.45E−02	**0.00E+00**	**0.00E+00**	1.24E−02	**0.00E+00**	**0.00E+00**	**0.00E+00**	1.63E+01
F_12_	Mean	**3.96E−09**	3.50E−02	1.16E+00	6.02E−01	7.34E+00	6.23E−03	5.59E−01	2.04E−01	3.08E−01	8.01E−03	7.66E−05	7.27E−01	5.53E+00
	Std	**6.16E−09**	2.15E−02	2.64E+00	6.35E−01	3.56E+00	1.94E−02	1.76E−01	6.73E−02	1.39E−01	6.09E−03	2.95E−05	1.19E−01	2.34E+00
F_13_	Mean	**8.99E−08**	5.05E−01	9.21E−01	3.52E−01	2.76E+00	8.10E−02	2.78E+00	1.65E+00	1.96E+00	2.31E−01	3.00E−01	2.89E+00	2.04E+00
	Std	**1.26E−07**	1.80E−01	2.34E+00	5.68E−01	6.62E−01	5.31E−02	3.42E−01	2.54E−01	1.45E−01	1.68E−01	9.07E−01	1.09E−01	4.91E+00
F_14_	Mean	1.78E+00	3.86E+00	2.08E+00	2.77E+00	7.47E+00	4.15E+00	2.97E+00	6.02E+00	**1.33E+00**	3.55E+00	1.03E+01	6.24E+00	3.91E+00
	Std	2.96E+00	4.43E+00	1.84E+00	2.22E+00	4.82E+00	2.91E+00	2.56E+00	4.27E+00	**7.52E−01**	3.76E+00	3.78E+00	4.62E+00	3.85E+00
F_15_	Mean	**3.55E−04**	5.78E−03	2.01E−03	2.59E−03	5.64E−03	8.82E−04	1.49E−03	2.50E−03	1.23E−03	6.20E−04	4.79E−04	4.91E−04	8.58E−03
	Std	**8.30E−10**	8.95E−03	3.74E−03	5.02E−03	1.47E−02	1.85E−04	3.67E−03	6.07E−03	2.80E−05	2.87E−04	4.25E−04	9.29E−05	1.18E−02
F_16_	Mean	**-1.03E+00**	**-1.03E+00**	**-1.03E+00**	**-1.03E+00**	**-1.03E+00**	**-1.03E+00**	**-1.03E+00**	**-1.03E+00**	**-1.03E+00**	**-1.03E+00**	-8.95E−01	**-1.03E+00**	-9.77E−01
	Std	**6.78E−16**	8.35E−09	**6.78E−16**	**6.78E−16**	1.29E−02	6.05E−15	6.98E−16	4.28E−05	5.64E−07	8.79E−11	3.09E−01	2.12E−05	2.07E−01
F_17_	Mean	**3.98E−01**	**3.98E−01**	3.97E−01	3.97E−01	**3.98E−01**	3.97E−01	4.49E−01	**3.98E−01**	5.53E−01	**3.98E−01**	3.97E−01	**3.98E−01**	3.97E−01
	Std	**0.00E+00**	6.58E−07	**0.00E+00**	**0.00E+00**	3.17E−05	**0.00E+00**	2.82E−01	1.78E−04	8.48E−01	4.80E−06	3.05E−03	2.52E−04	**0.00E+00**
F_18_	Mean	**3.00E+00**	**3.00E+00**	**3.00E+00**	**3.00E+00**	1.32E+01	**3.00E+00**	**3.00E+00**	**3.00E+00**	**3.00E+00**	**3.00E+00**	6.60E+00	**3.00E+00**	**3.00E+00**
	Std	1.05E−06	1.10E−05	1.71E−15	**1.31E−15**	2.59E+01	1.57E−15	5.55E−09	7.32E−06	4.23E−05	1.53E−05	9.34E+00	2.65E−06	1.43E−15
F_19_	Mean	**-3.86E+00**	**-3.86E+00**	**-3.86E+00**	**-3.86E+00**	**-3.86E+00**	**-3.86E+00**	-3.85E+00	-3.85E+00	-3.85E+00	-3.85E+00	**-3.86E+00**	-3.85E+00	-3.76E+00
	Std	**2.70E−15**	1.95E−03	2.71E−15	2.71E−15	1.99E−03	2.73E−15	4.45E−02	3.95E−03	2.60E−03	4.23E−03	1.78E−07	6.65E−03	2.67E−01
F_20_	Mean	**-3.29E+00**	-3.27E+00	-3.21E+00	-3.23E+00	-3.24E+00	-3.28E+00	-3.23E+00	-3.15E+00	-2.98E+00	-3.23E+00	-3.24E+00	-2.82E+00	-3.28E+00
	Std	**5.54E−02**	6.17E−02	8.48E−02	6.34E−02	6.79E−02	5.70E−02	1.12E−01	1.31E−01	3.00E−01	1.75E−01	5.75E−02	2.19E−01	5.60E−02
F_21_	Mean	**-10.1532**	-9.31E+00	-7.56E+00	-6.64E+00	-5.77E+00	-7.81E+00	-8.11E+00	-8.21E+00	-2.44E+00	-8.71E+00	-5.74E+00	-4.35E+00	-7.46E+00
	Std	**4.01E−05**	1.92E+00	3.32E+00	3.45E+00	3.36E+00	3.01E+00	2.53E+00	2.63E+00	3.45E+00	2.46E+00	1.76E+00	4.77E−01	3.03E+00
F_22_	Mean	**-10.4028**	-1.04E+01	-8.33E+00	-7.61E+00	-6.86E+00	-9.09E+00	-8.10E+00	-9.87E+00	-4.45E+00	-8.46E+00	-5.98E+00	-4.74E+00	-8.19E+00
	Std	**3.71E−05**	1.34E+00	3.26E+00	3.31E+00	3.50E+00	2.45E+00	3.02E+00	1.62E+00	4.46E+00	2.83E+00	2.01E+00	9.36E−01	3.24E+00
F_23_	Mean	**-10.5363**	-1.03E+01	-5.74E+00	-9.01E+00	-8.01E+00	-1.01E+01	-8.28E+00	-1.02E+01	-6.90E+00	-8.28E+00	-5.85E+00	-4.45E+00	-7.63E+00
	Std	**3.30E−05**	1.48E+00	3.54E+00	2.87E+00	3.61E+00	1.54E+00	3.20E+00	1.37E+00	4.17E+00	3.10E+00	1.87E+00	4.26E−01	3.66E+00

Significant are in value [bold].

The results show that the proposed IGWO outperforms other algorithms in terms of finding the optimal solution for single-peak and multimodal test functions, especially in terms of standard deviation, which has mostly improved by several orders of magnitude. It has achieved theoretical optimal values on functions *F*_1_–*F*_4_, *F*_9_, and *F*_11_, verifying that the implementation of the improvement strategy effectively improves the convergence accuracy and exploration and development capabilities of the GWO algorithm, providing more reliable solutions and technical support for solving complex optimization problems. For the multimodal problems with fixed dimensions (*F*_14_–*F*_23_), the IGWO proposed in this work performs better in *F*_15_–*F*_17_ and *F*_19_–*F*_23_. The reason why the IGWO algorithm performs suboptimal only on *F*_14_ and* F*_18_ is that it relies too much on searching near the individual's historical optimal position in the early stages of iteration. Although it improves the algorithm's global exploration ability and convergence speed, it lacks exploration around the optimal position, which to some extent limits the algorithm's potential to jump out of local optima.In summary, it can be concluded that the proposed IGWO algorithm has the best ability to explore the potential regions and avoid local optima in the search space.

**Figure 4:**
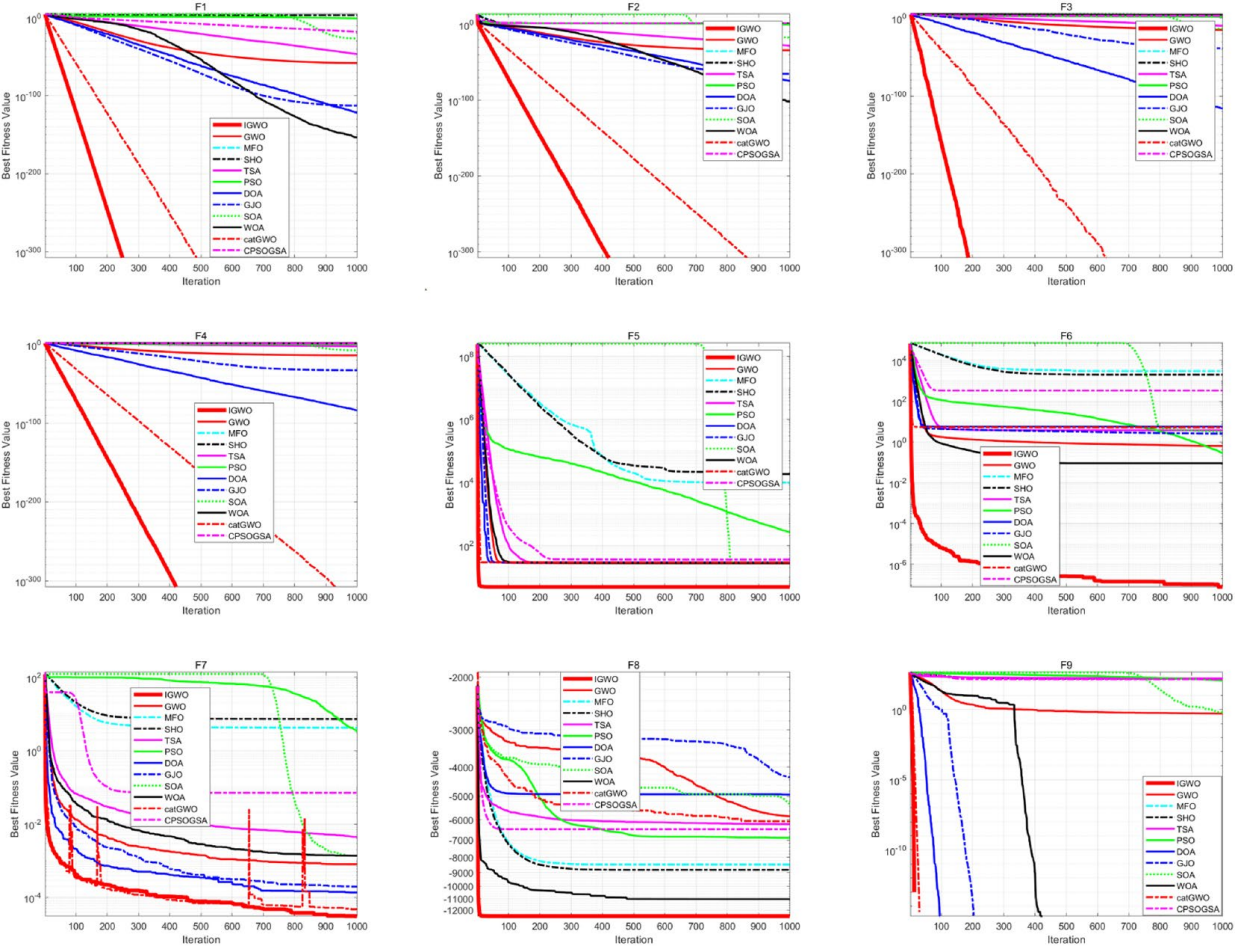

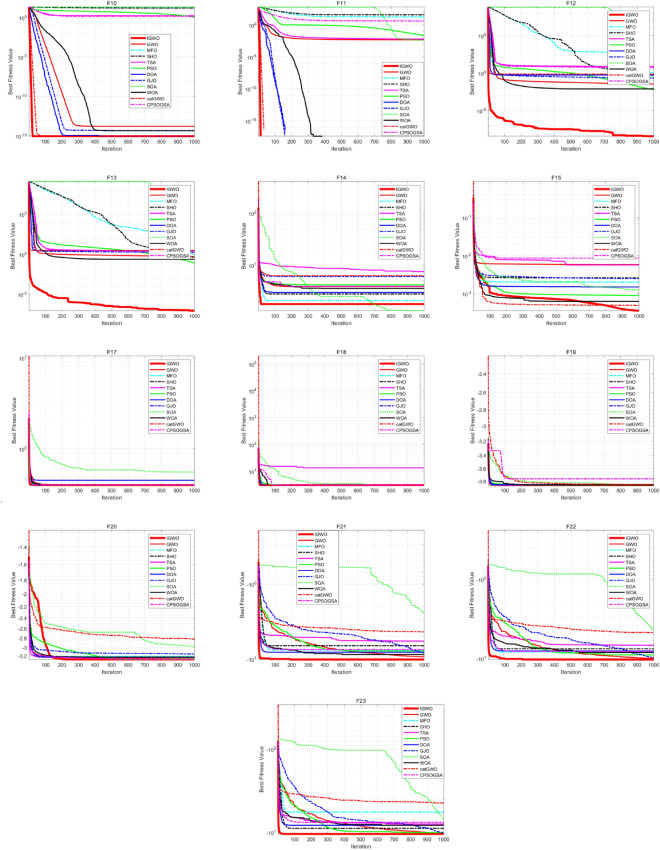
Convergence curve of the basis function.

In order to further analyze the performance of the proposed IGWO algorithm, the convergence curves of all the benchmark functions are shown in Fig. 4. The performance of the proposed IGWO algorithm is compared with that of GWO, MFO, SHO, TSA, PSO, DOA, GJO, SOA, WOA, catGWO, and CPSOGSA in this paper. It can be seen that the proposed IGWO algorithm outperforms other algorithms in terms of convergence speed and accuracy in most cases.

### Experiments on large-scale problems

In order to analyze the performance of the proposed IGWO algorithm on difficult optimization problems, numerical experiments are conducted on 100, 500, and 1000 dimensional problems as presented in scalable problems *F*_*1*_*-F*_*13*_, respectively. The IGWO algorithm is compared with GWO, MFO, SHO, TSA, PSO, DOA, GJO, SOA, WOA, catGWO, and CPSOGSA. For ensuring a fair comparison, all algorithms are executed under same conditions (i.e., the population size is set to 30 and the maximum number of iterations is set to 500). The results of all the experiments are averaged over 30 epochs. The specific results are shown in Table 6.

**Table 6:** Fitness results of different algorithms on 100, 500 and 1000 dimensional scalable benchmark problems.

Fun	Dim		IGWO	GWO	MFO	SHO	TSA	PSO	DOA	GJO	SOA	WOA	catGWO	CPSOGSA
F1	100	Mean	**0.00E+00**	1.60E−29	3.03E+04	3.69E+04	4.40E−25	8.76E+01	2.00E−116	2.76E−60	1.94E−15	1.13E−146	**0.00E+00**	1.01E+03
		Std	**0.00E+00**	1.55E−29	1.42E+04	1.48E+04	9.49E−25	1.65E+01	1.09E−115	7.68E−60	2.44E−15	6.06E−146	**0.00E+00**	1.77E+03
	500	Mean	**0.00E+00**	1.55E−12	9.58E+05	9.51E+05	9.08E−11	7.47E+03	2.64E−121	2.49E−32	6.00E−09	2.11E−144	**0.00E+00**	2.67E+05
		Std	**0.00E+00**	7.88E−13	3.83E+04	3.55E+04	9.08E−11	5.09E+02	1.43E−120	3.03E−32	5.74E−09	1.03E−143	**0.00E+00**	3.20E+04
	1000	Mean	**0.00E+00**	8.91E−09	2.47E+06	2.46E+06	5.75E−07	4.33E+04	2.50E−137	4.00E−26	3.81E−07	8.35E−144	**0.00E+00**	1.10E+06
		Std	**0.00E+00**	2.99E−09	4.58E+04	4.81E+04	6.16E−07	1.70E+03	1.37E−136	4.68E−26	9.30E−07	3.77E−143	**0.00E+00**	7.49E+04
F2	100	Mean	**0.00E+00**	5.64E−18	1.75E+02	1.77E+02	1.63E−16	1.24E+02	6.67E−73	5.59E−37	5.29E−11	8.21E−102	**0.00E+00**	3.54E+02
		Std	**0.00E+00**	2.85E−18	4.63E+01	4.96E+01	1.72E−16	4.45E+01	3.65E−72	4.72E−37	7.07E−11	4.25E−101	**0.00E+00**	3.30E+01
	500	Mean	**0.00E+00**	6.42E−08	2.26E+03	2.26E+03	1.88E−08	4.14E+133	2.31E−92	9.77E−21	1.27E−07	9.94E−101	**0.00E+00**	1.04E+266
		Std	**0.00E+00**	1.46E−08	8.00E+01	8.44E+01	1.18E−08	1.76E+134	1.25E−91	6.91E−21	8.40E−08	4.54E−100	**0.00E+00**	1.05E+267
	1000	Mean	**0.00E+00**	9.00E−05	2.53E+03	2.78E+03	5.17E−07	1.90E+221	4.10E−70	2.31E−17	7.11E−07	6.25E−101	5.63E−293	2.03E+326
		Std	**0.00E+00**	8.62E−05	1.00E+02	1.84E+02	3.03E−07	1.04E+222	2.24E−69	1.40E−17	4.59E−07	1.97E−100	**0.00E+00**	2.1E+347
F3	100	Mean	**0.00E+00**	6.72E+00	1.94E+05	1.99E+05	1.99E+03	1.42E+04	5.66E−112	8.88E−05	8.18E−01	9.69E+05	**0.00E+00**	8.29E+04
		Std	**0.00E+00**	1.23E+01	5.29E+04	4.91E+04	1.70E+03	3.66E+03	3.10E−111	4.83E−04	4.33E+00	2.10E+05	**0.00E+00**	1.63E+04
	500	Mean	**0.00E+00**	1.26E+05	3.50E+06	3.83E+06	1.08E+06	6.04E+05	1.37E−153	3.07E+03	3.05E+04	2.99E+07	**0.00E+00**	2.54E+06
		Std	**0.00E+00**	5.23E+04	5.94E+05	7.72E+05	1.88E+05	1.21E+05	7.51E−153	7.63E+03	4.55E+04	1.05E+07	**0.00E+00**	4.16E+05
	1000	Mean	**0.00E+00**	8.08E+05	1.42E+07	1.49E+07	4.63E+06	2.26E+06	5.12E−127	5.85E+04	2.23E+05	1.28E+08	**0.00E+00**	1.01E+07
		Std	**0.00E+00**	1.99E+05	2.27E+06	2.96E+06	6.51E+05	4.91E+05	2.81E−126	6.38E+04	2.99E+05	4.97E+07	**0.00E+00**	1.53E+06
F4	100	Mean	**0.00E+00**	6.23E−03	9.28E+01	9.35E+01	3.79E+01	1.03E+01	7.85E−74	1.00E−01	5.03E+01	8.00E+01	7.55E−319	7.16E+01
		Std	**0.00E+00**	1.83E−02	2.05E+00	1.61E+00	1.18E+01	1.25E+00	4.30E−73	2.30E−01	2.23E+01	1.84E+01	0.00E+00	1.62E+01
	500	Mean	**0.00E+00**	5.85E+01	9.90E+01	9.90E+01	9.92E+01	2.76E+01	2.02E−76	7.71E+01	9.81E+01	7.99E+01	7.85E−312	9.75E+01
		Std	**0.00E+00**	6.93E+00	2.60E−01	3.30E−01	2.80E−01	9.46E−01	9.82E−76	4.57E+00	5.37E−01	2.31E+01	0.00E+00	1.54E+00
	1000	Mean	**0.00E+00**	7.57E+01	9.95E+01	9.95E+01	9.96E+01	3.38E+01	6.63E−78	8.74E+01	9.94E+01	8.75E+01	6.49E−311	9.93E+01
		Std	**0.00E+00**	3.39E+00	1.79E−01	1.68E−01	1.24E−01	1.70E+00	3.63E−77	2.68E+00	2.17E−01	1.16E+01	**0.00E+00**	5.70E−01
F5	100	Mean	**6.53E+00**	9.74E+01	4.88E+07	6.75E+07	9.79E+01	9.08E+04	9.89E+01	9.82E+01	9.86E+01	9.78E+01	9.89E+01	2.34E+02
		Std	**2.49E+01**	9.15E−01	4.71E+07	5.36E+07	9.30E−01	1.60E+04	5.52E−02	6.88E−01	3.17E−01	3.58E−01	6.77E−02	8.32E+01
	500	Mean	**3.29E+01**	4.98E+02	4.02E+09	4.01E+09	4.98E+02	5.44E+07	4.99E+02	4.98E+02	4.99E+02	4.96E+02	4.99E+02	8.03E+07
		Std	**1.25E+02**	3.20E−01	2.62E+08	2.41E+08	2.20E−01	5.31E+06	3.50E−02	1.87E−01	1.47E−01	3.77E−01	5.98E−02	1.38E+07
	1000	Mean	**1.32E+02**	9.97E+02	1.10E+10	1.10E+10	8.30E+04	4.75E+08	9.99E+02	9.98E+02	9.99E+02	9.93E+02	9.99E+02	8.94E+08
		Std	3.42E+02	9.80E−02	3.56E+08	3.94E+08	6.34E+04	3.14E+07	**3.59E−02**	1.21E−01	1.67E−01	7.14E−01	5.22E−02	1.32E+08
F6	100	Mean	**1.17E−07**	9.60E+00	3.15E+04	3.68E+04	1.40E+01	9.96E+01	2.29E+01	1.69E+01	1.84E+01	2.08E+00	2.08E+00	1.40E+03
		Std	**1.78E−07**	8.37E−01	1.37E+04	1.27E+04	1.30E+00	1.63E+01	8.72E−01	9.25E−01	8.62E−01	6.24E−01	8.57E−01	2.39E+03
	500	Mean	**6.18E−07**	9.33E+01	9.58E+05	9.50E+05	9.57E+01	7.45E+03	1.23E+02	1.12E+02	1.16E+02	2.03E+01	1.18E+02	2.82E+05
		Std	**1.12E−06**	2.09E+00	3.24E+04	3.68E+04	2.27E+00	4.34E+02	9.98E−01	1.26E+00	9.88E−01	5.15E+00	1.71E+00	3.82E+04
	1000	Mean	**1.78E−06**	2.08E+02	2.47E+06	2.47E+06	2.12E+02	4.26E+04	2.48E+02	2.34E+02	2.40E+02	4.42E+01	2.43E+02	1.08E+06
		Std	**2.88E−06**	2.50E+00	5.82E+04	4.81E+04	1.72E+00	1.57E+03	1.10E+00	1.66E+00	8.42E−01	1.26E+01	1.70E+00	6.23E+04
F7	100	Mean	**3.19E−05**	2.87E−03	1.64E+02	1.78E+02	2.02E−02	1.36E+03	2.05E−04	4.93E−04	2.35E−03	2.20E−03	3.74E−05	1.20E+00
		Std	**2.68E−05**	1.37E−03	1.05E+02	1.23E+02	7.64E−03	2.31E+02	2.37E−04	3.31E−04	2.47E−03	3.15E−03	3.92E−05	2.82E−01
	500	Mean	**2.73E−05**	9.80E−03	2.98E+04	3.12E+04	4.79E−01	5.65E+04	1.41E−04	1.54E−03	1.06E−02	2.20E−03	3.99E−05	5.90E+02
		Std	**2.38E−05**	3.57E−03	2.56E+03	2.20E+03	1.86E−01	2.37E+03	1.95E−04	1.55E−03	4.25E−03	1.93E−03	3.70E−05	1.21E+02
	1000	Mean	**2.90E−05**	1.94E−02	1.75E+05	1.73E+05	3.81E+00	2.41E+05	1.19E−04	2.53E−03	2.38E−02	1.85E−03	3.89E−05	1.15E+04
		Std	**2.18E−05**	5.28E−03	5.58E+03	5.55E+03	1.20E+00	5.10E+03	1.58E−04	1.41E−03	1.06E−02	2.35E−03	3.01E−05	1.62E+03
F8	100	Mean	**-4.19E+04**	-1.62E+04	-2.44E+04	-2.37E+04	-1.34E+04	-2.18E+04	-9.07E+03	-1.08E+04	-1.10E+04	-3.78E+04	-1.17E+04	-1.83E+04
		Std	**1.66E-02**	2.13E+03	2.95E+03	2.39E+03	9.02E+02	2.40E+03	1.34E+03	4.14E+03	2.71E+03	5.12E+03	3.06E+03	1.23E+03
	500	Mean	**-2.09E+05**	-6.36E+04	-7.35E+04	-7.12E+04	-3.30E+04	-1.08E+05	-2.04E+04	-2.91E+04	-2.57E+04	-1.98E+05	-2.81E+04	-4.67E+04
		Std	**1.42E−01**	4.62E+03	5.89E+03	4.81E+03	1.71E+03	1.02E+04	3.41E+03	1.49E+04	5.55E+03	1.46E+04	6.79E+03	2.83E+03
	1000	Mean	**-4.19E+05**	-9.86E+04	-1.07E+05	-1.08E+05	-4.63E+04	-1.67E+05	-2.89E+04	-4.25E+04	-3.75E+04	-3.87E+05	-3.90E+04	-7.07E+04
		Std	**8.75E−02**	1.62E+04	5.67E+03	7.64E+03	2.96E+03	5.86E+04	7.56E+03	2.62E+04	8.84E+03	4.51E+04	1.07E+04	4.82E+03
F9	100	Mean	**0.00E+00**	1.37E+00	7.37E+02	7.46E+02	9.35E+02	1.05E+03	**0.00E+00**	**0.00E+00**	2.96E−13	**0.00E+00**	**0.00E+00**	4.82E+02
		Std	**0.00E+00**	2.48E+00	6.80E+01	9.89E+01	1.56E+02	1.23E+02	**0.00E+00**	**0.00E+00**	4.47E−13	**0.00E+00**	**0.00E+00**	5.87E+01
	500	Mean	**0.00E+00**	5.41E+00	6.42E+03	6.47E+03	5.42E+03	7.88E+03	**0.00E+00**	9.09E−14	5.65E−01	6.06E−14	**0.00E+00**	3.14E+03
		Std	**0.00E+00**	6.18E+00	1.11E+02	1.53E+02	7.90E+02	3.03E+02	**0.00E+00**	2.78E−13	2.50E+00	3.32E−13	**0.00E+00**	2.84E+02
	1000	Mean	**0.00E+00**	1.22E+01	1.47E+04	1.48E+04	9.56E+03	1.66E+04	0.00E+00	6.06E−14	5.68E−01	**0.00E+00**	**0.00E+00**	8.65E+03
		Std	**0.00E+00**	8.22E+00	2.15E+02	2.12E+02	2.13E+03	3.54E+02	0.00E+00	3.32E−13	1.63E+00	**0.00E+00**	**0.00E+00**	6.00E+02
F10	100	Mean	**8.88E−16**	1.12E−13	1.98E+01	1.99E+01	4.56E−13	5.43E+00	1.01E−15	9.41E−15	2.00E+01	3.61E−15	**8.88E−16**	1.68E+01
		Std	**0.00E+00**	8.71E−15	2.24E−01	1.77E−01	1.06E−12	3.54E−01	6.49E−16	2.57E−15	3.65E−04	2.22E−15	**0.00E+00**	2.66E+00
	500	Mean	**8.88E−16**	5.32E−08	2.01E+01	2.01E+01	4.93E−07	1.29E+01	8.88E−16	3.81E−14	2.00E+01	4.20E−15	**8.88E−16**	1.96E+01
		Std	**0.00E+00**	1.29E−08	1.25E−01	1.34E−01	2.17E−07	2.33E−01	0.00E+00	3.93E−15	8.84E−05	2.79E−15	**0.00E+00**	1.68E−01
	1000	Mean	**8.88E−16**	3.21E−06	2.01E+01	2.01E+01	2.17E−05	1.65E+01	1.01E−15	8.64E−14	2.00E+01	4.56E−15	**8.88E−16**	1.99E+01
		Std	**0.00E+00**	5.90E−07	2.04E−01	1.82E−01	1.10E−05	2.30E−01	6.49E−16	1.15E−14	4.52E−05	2.38E−15	**0.00E+00**	1.77E−01
F11	100	Mean	**0.00E+00**	**0.00E+00**	3.12E+02	2.96E+02	6.39E−03	7.88E−01	**0.00E+00**	**0.00E+00**	3.85E−03	8.79E−03	**0.00E+00**	1.30E+02
		Std	**0.00E+00**	**0.00E+00**	1.27E+02	7.17E+01	9.40E−03	8.96E−02	**0.00E+00**	**0.00E+00**	1.20E−02	3.37E−02	**0.00E+00**	6.02E+01
	500	Mean	**0.00E+00**	1.58E−03	8.63E+03	8.65E+03	8.51E−03	3.49E+00	**0.00E+00**	6.29E−17	5.64E−03	7.15E−03	**0.00E+00**	4.69E+03
		Std	**0.00E+00**	6.01E−03	3.64E+02	3.15E+02	2.00E−02	1.55E−01	**0.00E+00**	5.60E−17	2.28E−02	3.92E−02	**0.00E+00**	3.41E+02
	1000	Mean	**0.00E+00**	1.32E−03	2.22E+04	2.23E+04	1.68E−02	2.92E+01	**0.00E+00**	1.41E−16	3.60E−08	7.40E−18	**0.00E+00**	1.30E+04
		Std	**0.00E+00**	7.21E−03	4.17E+02	4.90E+02	3.61E−02	2.04E+00	**0.00E+00**	4.99E−17	6.62E−08	2.82E−17	**0.00E+00**	6.56E+02
F12	100	Mean	**1.64E−09**	2.34E−01	1.04E+08	9.05E+07	1.16E+01	5.84E+00	9.57E−01	6.36E−01	7.36E−01	1.58E−02	1.01E+00	1.93E+01
		Std	**4.21E−09**	5.21E−02	1.36E+08	1.08E+08	4.47E+00	2.59E+00	1.06E−01	7.75E−02	9.44E−02	5.55E−03	5.95E−02	3.66E+00
	500	Mean	**7.66E−10**	7.46E−01	9.55E+09	9.57E+09	5.24E+04	7.98E+05	1.16E+00	9.59E−01	1.02E+00	4.27E−02	1.12E+00	8.49E+07
		Std	**9.54E−10**	2.92E−02	6.79E+08	7.69E+08	7.48E+04	3.29E+05	2.38E−02	2.68E−02	2.12E−02	1.99E−02	2.43E−02	2.13E+07
	1000	Mean	**1.87E−09**	8.60E−01	2.70E+10	2.69E+10	1.63E+07	2.24E+07	1.16E+00	1.03E+00	1.09E+00	4.50E−02	1.14E+00	1.13E+09
		Std	**4.36E−09**	2.43E−02	8.96E+08	1.08E+09	1.28E+07	3.74E+06	1.53E−02	1.61E−02	2.64E−02	1.78E−02	1.42E−02	2.13E+08
F13	100	Mean	**1.28E−07**	6.19E+00	2.96E+08	2.72E+08	1.24E+01	9.17E+01	9.99E+00	8.62E+00	8.93E+00	1.58E+00	9.97E+00	4.79E+02
		Std	**2.15E−07**	6.08E−01	2.76E+08	2.51E+08	1.53E+00	2.68E+01	4.34E−03	2.57E−01	2.34E−01	6.63E−01	3.00E−02	4.35E+02
	500	Mean	**4.41E−07**	4.61E+01	1.81E+10	1.77E+10	5.81E+03	8.14E+06	5.00E+01	4.82E+01	4.95E+01	1.06E+01	5.00E+01	3.79E+08
		Std	**8.98E−07**	4.36E−01	1.20E+09	1.31E+09	4.71E+03	1.46E+06	3.83E−03	3.54E−01	6.33E−01	4.29E+00	1.60E−02	1.15E+08
	1000	Mean	**4.92E−07**	9.61E+01	4.96E+10	4.96E+10	3.42E+06	1.58E+08	1.00E+02	9.80E+01	1.01E+02	2.43E+01	1.00E+02	3.51E+09
		Std	**1.01E−06**	6.53E−01	1.72E+09	1.73E+09	2.98E+06	1.92E+07	5.13E−03	3.61E−01	1.05E+00	1.03E+01	2.59E−02	4.58E+08

Significant are in value [bold].

**Figure 5:**
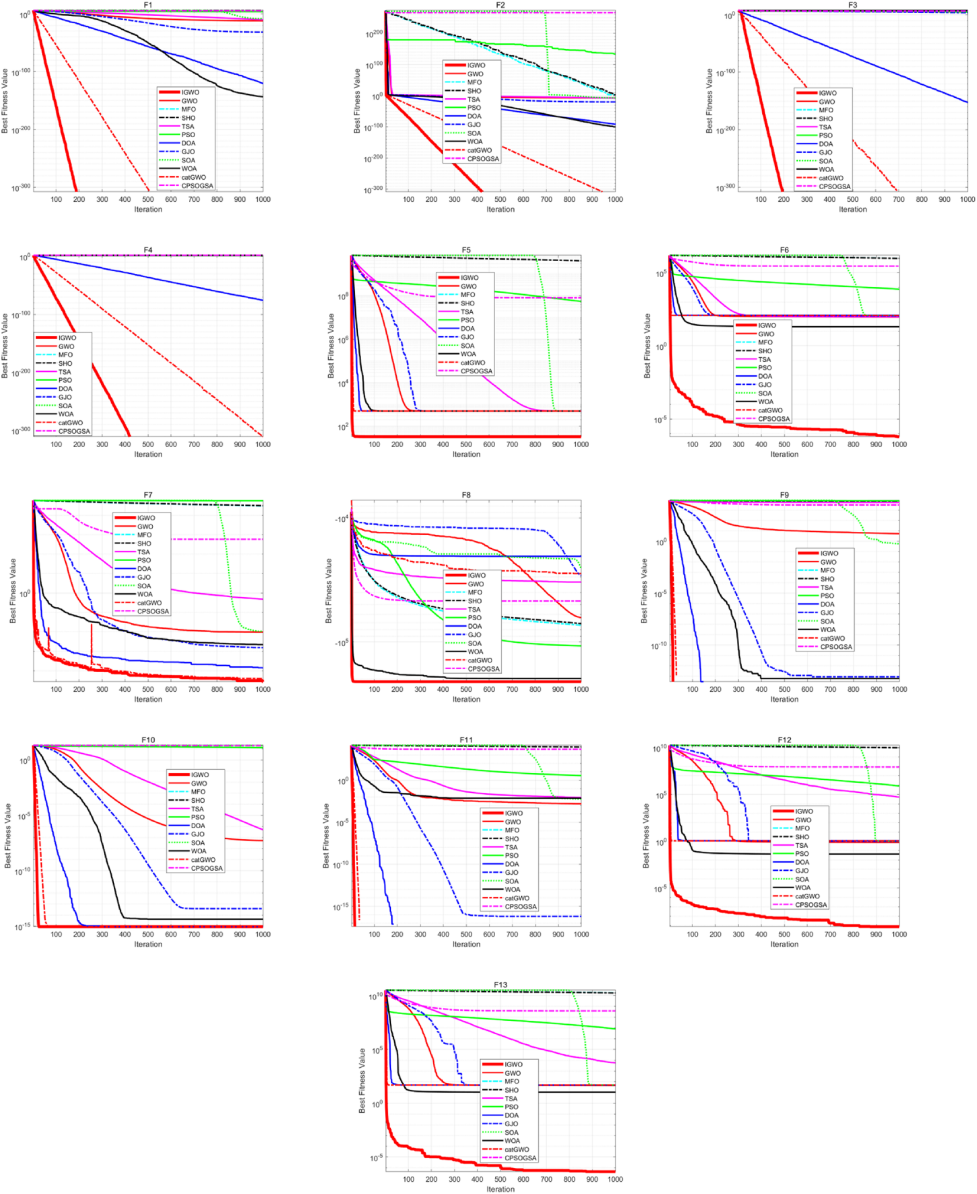
Convergence curves on 500 dimensional problems.

From Table 6, it can be seen that for 100, 500, and 1000 dimensional problems, the IGWO algorithm proposed in this paper has better performance as compared to other algorithms. In addition, as the dimensionality increases, the proposed IGWO continues to provide the best solution. Therefore, it can be concluded that the proposed IGWO is insensitive to the growth of dimensionality and has superior optimization search capability and scalability. Figure 5 depicts the convergence curves of the algorithms under 500 dimensions. From these figures, it can be seen that the proposed IGWO converges faster while maintaining a higher level of dimensional accuracy as compared to other swarm intelligence optimization algorithms. The aforementioned analysis shows that the adopted strategy is suitable for complex problems and IGWO performs more robustly for higher dimensional problems.

### Statistical analysis

In order to determine whether the proposed IGWO algorithm is significantly better than its competitors, this paper uses a nonparametric test, i.e., the Wilcoxon rank sum test^46^, for 30, 100, 500, and 1000 dimensions. The statistical results at 5% significance level are shown in Tables 7 and 8. In the tables, + /−/≈ denote that IGWO is better/worse/same as the algorithm being compared, respectively.

**Table 7:** Wilcoxon rank sum test statistics for 23 benchmark functions.

Fun		GWO	MFO	SHO	TSA	PSO	DOA	GJO	SOA	WOA	catGWO	CPSOGSA
F1	*p*-value	1.21E−12	1.21E−12	1.21E−12	1.21E−12	1.21E−12	8.87E−07	1.21E−12	1.21E−12	1.21E−12	NaN	1.21E−12
	Outcome	+	+	+	+	+	+	+	+	+	=	+
F2	*p*-value	1.21E−12	1.21E−12	1.21E−12	1.21E−12	1.21E−12	1.93E−10	1.21E−12	1.21E−12	1.21E−12	NaN	1.21E−12
	Outcome	+	+	+	+	+	+	+	+	+	=	+
F3	*p*-value	1.21E−12	1.21E−12	1.21E−12	1.21E−12	1.21E−12	6.61E−05	1.21E−12	1.21E−12	1.21E−12	NaN	1.21E−12
	Outcome	+	+	+	+	+	+	+	+	+	=	+
F4	*p*-value	1.21E−12	1.21E−12	1.21E−12	1.21E−12	1.21E−12	1.93E−10	1.21E−12	1.21E−12	1.21E−12	NaN	1.21E−12
	Outcome	+	+	+	+	+	+	+	+	+	=	+
F5	*p*-value	6.74E−06	8.15E−11	8.15E−11	1.07E−07	3.02E−11	3.02E−11	4.74E−06	1.11E−06	3.32E−06	8.89E−10	2.38E−07
	Outcome	+	+	+	+	+	+	+	+	+	+	+
F6	*p*-value	3.02E−11	3.02E−11	3.02E−11	3.02E−11	3.02E−11	3.02E−11	3.02E−11	3.02E−11	3.02E−11	3.02E−11	5.57E−10
	Outcome	+	+	+	+	+	+	+	+	+	+	+
F7	*p*-value	3.02E−11	3.02E−11	3.02E−11	3.02E−11	3.02E−11	1.87E−05	5.97E−09	3.02E−11	4.98E−11	2.22E−01	3.02E−11
	Outcome	+	+	+	+	+	+	+	+	+	+	+
F8	*p*-value	3.02E−11	3.02E−11	3.02E−11	3.02E−11	3.02E−11	3.02E−11	3.02E−11	3.02E−11	3.02E−11	3.02E−11	3.02E−11
	Outcome	+	+	+	+	+	+	+	+	+	+	+
F9	*p*-value	5.58E−03	1.21E−12	1.21E−12	1.21E−12	1.21E−12	3.78E+00	3.78E+00	1.10E−02	3.78E+00	3.78E+00	1.21E−12
	Outcome	+	+	+	+	+	=	=	+	=	=	+
F10	*p*-value	1.59E−13	1.21E−12	1.21E−12	1.09E−12	1.21E−12	NaN	2.71E−14	1.21E−12	3.36E−09	NaN	1.21E−12
	Outcome	+	+	+	+	+	=	+	+	+	=	+
F11	*p*-value	5.58E−03	1.21E−12	1.21E−12	2.93E−05	1.21E−12	NaN	NaN	8.15E−02	NaN	NaN	1.21E−12
	Outcome	+	+	+	+	+	=	=	+	=	=	+
F12	*p*-value	3.02E−11	3.02E−11	3.02E−11	3.02E−11	3.02E−11	3.02E−11	3.02E−11	3.02E−11	3.02E−11	3.02E−11	3.02E−11
	Outcome	+	+	+	+	+	+	+	+	+	+	+
F13	*p*-value	3.02E−11	3.02E−11	3.02E−11	3.02E−11	3.02E−11	3.02E−11	3.02E−11	3.02E−11	3.02E−11	3.02E−11	3.79E−01
	Outcome	+	+	+	+	+	+	+	+	+	+	+
F14	*p*-value	2.43E−05	8.89E−02	2.27E−01	1.20E−08	1.42E−02	2.22E−01	5.96E−09	1.01E−08	9.52E−04	2.03E−09	9.27E−02
	Outcome	+	+	+	+	+	+	+	-	+	+	+
F15	*p*-value	5.30E−01	9.78E−05	2.12E−04	1.68E−03	1.77E−03	1.15E−01	1.26E−01	9.51E−06	2.16E−03	2.89E−03	1.17E−03
	Outcome	+	+	+	+	+	+	+	+	+	+	+
F16	*p*-value	7.96E−01	1.21E−12	1.21E−12	9.92E−11	1.25E−11	1.34E−11	2.38E−07	2.37E−10	3.02E−11	3.02E−11	5.41E−09
	Outcome	=	+	+	+	+	+	+	+	+	+	+
	*p*-value	3.11E−01	1.21E−12	1.21E−12	5.60E−07	1.21E−12	6.03E−11	2.96E−05	4.31E−08	4.84E−02	2.44E−09	1.21E−12
	Outcome	+	+	+	+	+	+	+	+	+	+	+
F17	*p*-value	9.93E−02	2.36E−11	2.14E−11	4.83E−01	2.35E−11	2.35E−11	2.83E−08	6.00E−01	9.35E−01	5.26E−04	2.70E−11
	Outcome	+	+	+	-	+	+	+	+	+	+	+
F19	*p*-value	5.11E−01	1.21E−12	1.21E−12	2.97E−01	6.32E−12	1.81E−07	7.96E−03	9.76E−10	1.24E−03	8.99E−11	8.52E−07
	Outcome	+	+	+	+	+	+	+	+	+	+	+
F20	*p*-value	1.67E−01	9.89E−02	3.29E−01	2.15E−06	6.73E−04	2.84E−04	3.20E−09	4.08E−11	1.17E−05	3.02E−11	1.51E−04
	*o*utcome	+	+	+	+	+	+	+	+	+	+	+
F21	*p*-value	6.07E−11	1.80E−01	6.60E−01	3.02E−11	1.82E−01	6.83E−01	3.02E−11	3.02E−11	4.08E−11	3.02E−11	6.61E−01
	Outcome	+	+	+	+	+	+	+	+	+	+	+
F22	*p*-value	2.37E−10	7.51E−03	3.78E−01	3.02E−11	2.95E−04	6.51E−01	3.02E−11	3.02E−11	3.02E−11	3.02E−11	2.54E−02
	Outcome	+	+	+	+	+	+	+	+	+	+	+
F23	*p*-value	8.10E−10	2.67E−02	3.39E−04	3.02E−11	4.38E−09	3.77E−01	3.02E−11	3.02E−11	1.55E−09	3.02E−11	1.82E−01
	Outcome	+	+	+	+	+	+	+	+	+	+	+

**Table 8:** Statistical results of Wilcoxon rank sum test for benchmark functions on 100, 500 and 1000 dimensions.

Fun	Dim		GWO	MFO	SHO	TSA	PSO	DOA	GJO	SOA	WOA	catGWO	CPSOGSA
F1	100	*p*-value	1.21E−12	1.21E−12	1.21E−12	1.21E−12	1.21E−12	8.87E−07	1.21E−12	1.21E−12	1.21E−12	1.70E+00	1.21E−12
		Outcome	+	+	+	+	+	+	+	+	+	=	+
	500	p*-*value	1.21E−12	1.21E−12	1.21E−12	1.21E−12	1.21E−12	1.46E−04	1.21E−12	1.21E−12	1.21E−12	1.80E+00	1.21E−12
		Outcome	+	+	+	+	+	+	+	+	+	=	+
	1000	*p*-value	1.21E−12	1.21E−12	1.21E−12	1.21E−12	1.21E−12	8.87E−07	1.21E−12	1.21E−12	1.21E−12	NaN	1.21E−12
		Outcome	+	+	+	+	+	+	+	+	+	=	+
F2	100	*p*-value	1.21E−12	1.21E−12	1.21E−12	1.21E−12	1.21E−12	1.66E−11	1.21E−12	1.21E−12	1.21E−12	NaN	1.21E−12
		Outcome	+	+	+	+	+	+	+	+	+	=	+
	500	*p*-value	1.21E−12	1.21E−12	1.21E−12	1.21E−12	1.21E−12	5.85E−09	1.21E−12	1.21E−12	1.21E−12	NaN	1.21E−12
		Outcome	+	+	+	+	+	+	+	+	+	=	+
	1000	*p*-value	1.21E−12	NaN	NaN	1.21E−12	1.21E−12	1.21E−12	1.21E−12	1.21E−12	1.21E−12	NaN	NaN
		Outcome	+	=	=	+	+	+	+	+	+	=	=
F3	100	*p*-value	1.21E-12	1.21E-12	1.21E-12	1.21E-12	1.21E-12	2.93E-05	1.21E-12	1.21E-12	1.21E-12	NaN	1.21E-12
		Outcome	+	+	+	+	+	+	+	+	+	=	+
	500	*p*-value	1.21E-12	1.21E−12	1.21E−12	1.21E−12	1.21E−12	2.21E−06	1.21E−12	1.21E−12	1.21E−12	1.72E+00	1.21E−12
		Outcome	+	+	+	+	+	+	+	+	+	=	+
	1000	*p*-value	1.21E−12	1.21E−12	1.21E−12	1.21E−12	1.21E−12	3.45E−07	1.21E−12	1.21E−12	1.21E−12	NaN	1.21E−12
		Outcome	+	+	+	+	+	+	+	+	+	=	+
F4	100	*p*-value	1.21E−12	1.21E−12	1.21E−12	1.21E−12	1.21E−12	5.77E−11	1.21E−12	1.21E−12	1.21E−12	1.70E−08	1.21E−12
		Outcome	+	+	+	+	+	+	+	+	+	+	+
	500	*p*-value	1.21E−12	1.21E−12	1.21E−12	1.21E−12	1.21E−12	1.95E−09	1.21E−12	1.21E−12	1.21E−12	1.21E−12	1.21E−12
		Outcome	+	+	+	+	+	+	+	+	+	+	+
	1000	*p*-value	1.21E−12	1.21E−12	1.21E−12	1.21E−12	1.21E−12	1.66E−11	1.21E−12	1.21E−12	1.21E−12	1.21E−12	1.21E−12
		Outcome	+	+	+	+	+	+	+	+	+	+	+
F5	100	*p*-value	1.17E−09	3.02E−11	3.02E−11	3.16E−10	3.02E−11	3.02E−11	2.15E−10	3.69E−11	1.17E−09	3.02E−11	4.50E−11
		Outcome	+	+	+	+	+	+	+	+	+	+	+
	500	*p*-value	3.02E−11	3.02E−11	3.02E−11	3.02E−11	3.02E−11	3.02E−11	3.02E−11	3.02E−11	3.02E−11	3.02E−11	3.02E−11
		Outcome	+	+	+	+	+	+	+	+	+	+	+
	1000	*p*-value	3.02E−11	3.02E−11	3.02E−11	3.02E−11	3.02E−11	3.02E−11	3.02E−11	3.02E−11	3.02E−11	3.02E−11	3.02E−11
		Outcome	+	+	+	+	+	+	+	+	+	+	+
F6	100	*p*-value	3.02E−11	3.02E−11	3.02E−11	3.02E−11	3.02E−11	3.02E−11	3.02E−11	3.02E−11	3.02E−11	3.02E−11	3.02E−11
		Outcome	+	+	+	+	+	+	+	+	+	+	+
	500	*p*-value	3.02E−11	3.02E−11	3.02E−11	3.02E−11	3.02E−11	3.02E−11	3.02E−11	3.02E−11	3.02E−11	3.02E−11	3.02E−11
		Outcome	+	+	+	+	+	+	+	+	+	+	+
	1000	*p*-value	3.02E−11	3.02E−11	3.02E−11	3.02E−11	3.02E−11	3.02E−11	3.02E−11	3.02E−11	3.02E−11	3.02E−11	3.02E−11
		Outcome	+	+	+	+	+	+	+	+	+	+	+
F7	100	*p*-value	3.02E−11	3.02E−11	3.02E−11	3.02E−11	3.02E−11	1.75E−05	3.02E−11	6.70E−11	6.70E−11	8.07E−01	3.02E−11
		Outcome	+	+	+	+	+	+	+	+	+	+	+
	500	*p*-value	3.02E−11	3.02E−11	3.02E−11	3.02E−11	3.02E−11	2.32E−06	3.02E−11	3.02E−11	8.99E−11	1.96E−01	3.02E−11
		Outcome	+	+	+	+	+	+	+	+	+	+	+
	1000	*p*-value	3.02E−11	3.02E−11	3.02E−11	3.02E−11	3.02E−11	5.61E−05	3.02E−11	3.02E−11	3.02E−11	2.71E−01	3.02E−11
		Outcome	+	+	+	+	+	+	+	+	+	+	+
F8	100	*p*-value	3.02E−11	3.02E−11	3.02E−11	3.02E−11	3.02E−11	3.02E−11	3.02E−11	3.02E−11	3.02E−11	3.02E−11	3.02E−11
		Outcome	+	+	+	+	+	+	+	+	+	+	+
	500	*p*-value	3.02E−11	3.02E−11	3.02E−11	3.02E−11	3.02E−11	3.02E−11	3.02E−11	3.02E−11	3.02E−11	3.02E−11	3.02E−11
		Outcome	+	+	+	+	+	+	+	+	+	+	+
	1000	*p*-value	3.02E−11	3.02E−11	3.02E−11	3.02E−11	3.02E−11	3.02E−11	3.02E−11	3.02E−11	3.02E−11	3.02E−11	3.02E−11
		Outcome	+	+	+	+	+	+	+	+	+	+	+
F9	100	*p*-value	1.37E−11	1.21E−12	1.21E−12	1.21E−12	1.21E−12	NaN	NaN	5.37E−10	NaN	NaN	1.21E−12
		Outcome	+	+	+	+	+	=	=	+	=	=	+
	500	*p*-value	1.21E−12	1.21E−12	1.21E−12	1.21E−12	1.21E−12	NaN	8.14E−02	1.21E−12	3.34E−01	NaN	1.21E−12
		Outcome	+	+	+	+	+	=	+	+	+	=	+
	1000	*p*-value	1.21E−12	1.21E−12	1.21E−12	1.21E−12	1.21E−12	NaN	3.34E−01	1.21E−12	NaN	NaN	1.21E−12
		Outcome	+	+	+	+	+	=	+	+	+	=	+
F10	100	*p*-value	1.02E−12	1.21E−12	1.21E−12	1.21E−12	1.21E−12	3.34E−01	2.57E−13	1.21E−12	8.07E−08	NaN	1.21E−12
		Outcome	+	+	+	+	+	+	+	+	+	=	+
	500	*p*-value	1.21E−12	1.21E−12	1.21E−12	1.21E−12	1.21E−12	NaN	1.21E−12	1.21E−12	1.21E−12	NaN	1.21E−12
		Outcome	+	+	+	+	+	=	+	+	+	=	+
	1000	*p*-value	1.21E−12	1.21E−12	1.21E−12	1.21E−12	1.21E−12	3.34E−01	1.21E−12	9.57E−13	1.08E−09	NaN	1.21E−12
		Outcome	+	+	+	+	+	+	+	+	+	=	+
F11	100	*p*-value	NaN	1.21E−12	1.21E−12	5.37E−06	1.21E−12	NaN	NaN	1.20E−12	8.15E−02	NaN	1.21E−12
		Outcome	=	+	+	+	+	=	=	+	+	=	+
	500	*p*-value	1.21E−12	1.21E−12	1.21E−12	1.21E−12	1.21E−12	NaN	1.43E−06	1.21E−12	3.34E−01	NaN	1.21E−12
		Outcome	+	+	+	+	+	=	+	+	+	=	+
	1000	*p*-value	1.21E−12	1.21E−12	1.21E−12	1.21E−12	1.21E−12	NaN	2.43E−13	1.21E−12	1.61E−01	NaN	1.21E−12
		Outcome	+	+	+	+	+	=	+	+	+	=	+
F12	100	*p*-value	3.02E−11	3.02E−11	3.02E−11	3.02E−11	3.02E−11	3.02E−11	3.02E−11	3.02E−11	3.02E−11	3.02E−11	3.02E−11
		Outcome	+	+	+	+	+	+	+	+	+	+	+
	500	*p*-value	3.02E−11	3.02E−11	3.02E−11	3.02E−11	3.02E−11	3.02E−11	3.02E−11	3.02E−11	3.02E−11	3.02E−11	3.02E−11
		Outcome	+	+	+	+	+	+	+	+	+	+	+
	1000	*p*-value	3.02E−11	3.02E−11	3.02E−11	3.02E−11	3.02E−11	3.02E−11	3.02E−11	3.02E−11	3.02E−11	3.02E−11	3.02E−11
		Outcome	+	+	+	+	+	+	+	+	+	+	+
F13	100	*p*-value	3.02E−11	3.02E−11	3.02E−11	3.02E−11	3.02E−11	3.02E−11	3.02E−11	3.02E−11	3.02E−11	3.02E−11	3.02E−11
		Outcome	+	+	+	+	+	+	+	+	+	+	+
	500	*p*-value	3.02E−11	3.02E−11	3.02E−11	3.02E−11	3.02E−11	3.02E−11	3.02E−11	3.02E−11	3.02E−11	3.02E−11	3.02E−11
		Outcome	+	+	+	+	+	+	+	+	+	+	+
	1000	*p*-value	3.02E−11	3.02E−11	3.02E−11	3.02E−11	3.02E−11	3.02E−11	3.02E−11	3.02E−11	3.02E−11	3.02E−11	3.02E−11
		Outcome	+	+	+	+	+	+	+	+	+	+	+

Friedman test is a nonparametric and multiple comparison test used to assess significant differences between two or more algorithms. In this work, it is used to assess significant differences in five different dimensions (Dim = 30, 100, 500, and 1000)^47^. Table 9 shows the statistical results for four different dimensions, which are sorted based on the average ranking produced by the Friedman test. It is noteworthy that the proposed IGWO consistently performs best in different dimensions.

**Table 9:** The average ranking of the algorithm is obtained by Friedman's test.

30 Dimensional		100 Dimensional		500 Dimensional		1000 Dimensional	
**IGWO**	**1.91**	**IGWO**	**1.69**	**IGWO**	**1.59**	**IGWO**	**1.50**
GWO	5.58	GWO	4.98	GWO	5.21	GWO	5.17
MFO	9.03	MFO	10.37	MFO	10.56	MFO	10.52
SHO	8.85	SHO	10.47	SHO	10.56	SHO	10.54
TSA	8.04	TSA	7.18	TSA	7.41	TSA	7.66
PSO	8.11	PSO	8.77	PSO	8.62	PSO	8.37
DOA	5.63	DOA	4.84	DOA	4.78	DOA	4.54
GJO	5.46	GJO	4.80	GJO	4.98	GJO	4.93
SOA	7.32	SOA	7.05	SOA	7.11	SOA	6.93
WOA	5.04	WOA	4.36	WOA	3.92	WOA	3.77
catGWO	4.96	catGWO	4.19	catGWO	4.03	catGWO	3.84
CPSOGSA	8.07	CPSOGSA	9.28	CPSOGSA	9.25	CPSOGSA	9.51
*p*-value	7.74E−47	*p*-value	9.18E−59	*p*-value	2.83E−60	*p*-value	2.40E−55

Significant are in value [bold].

### Comparison with other algorithms on CEC2014 benchmark functions

In this section, 15 benchmark functions from CEC2014 are used to further evaluate the performance of the proposed IGWO algorithm. The names, classes, and global optima of the functions are presented in Table 2. The dimensions and ranges of the variables are 10 and [− 100, 100], respectively. The chosen benchmark function with constraints and high computational cost is challenging as compared to the classical benchmark function.

The functions in CEC2014 provide complex shapes and multiple local optima that approximate the search space of real-world problems, thus effectively evaluating the effectiveness and robustness of the model. Table 10 shows the average fitness values (Avg) and standard deviation (Std) of fitness values obtained by different algorithms for solving the CEC2014 problem. As presented in Table 10, the proposed IGWO ranks first in terms of fitness values on 15 CEC2014 test functions. As compared with the original GWO algorithm, the proposed IGWO algorithm shows a significant improvement in the optimality searching ability on 10 functions, and the average fitness value for the remaining 5 functions is the same as that of the original GWO algorithm. In addition, as compared with other 11 algorithms, although the solutions of some algorithms on individual functions are equal to or slightly better than IGWO, in general, IGWO outperforms the other algorithms on other functions. In summary, IGWO has better optimization seeking performance as compared to GWO in dealing with complex optimization problems.

**Table 10:** Fitness results of different algorithms on 15 CEC2014 benchmark functions.

Fun		IGWO	GWO	MFO	SHO	TSA	PSO	DOA	GJO	SOA	WOA	catGWO	CPSOGSA
F03	Mean	**2.21E+03**	8.08E+03	1.84E+04	4.24E+05	1.23E+04	6.11E+03	9.51E+03	6.63E+03	6.57E+03	5.34E+04	1.60E+04	1.24E+04
	Std	**2.86E+03**	4.43E+03	1.41E+04	8.33E+05	4.12E+03	4.18E+03	5.53E+03	3.71E+03	3.88E+03	2.30E+04	1.97E+03	7.69E+03
F06	Mean	**6.02E+02**	6.03E+02	6.05E+02	6.11E+02	6.07E+02	6.03E+02	6.07E+02	6.05E+02	6.07E+02	6.08E+02	6.11E+02	6.04E+02
	Std	**1.19E+00**	1.27E+00	1.94E+00	1.20E+00	2.19E+00	1.87E+00	1.43E+00	1.44E+00	1.36E+00	2.05E+00	7.68E−01	2.06E+00
F08	Mean	**8.12E+02**	8.13E+02	8.21E+02	9.22E+02	8.41E+02	8.26E+02	8.48E+02	8.30E+02	8.26E+02	8.40E+02	8.77E+02	8.34E+02
	Std	**5.81E+00**	6.90E+00	8.50E+00	1.89E+01	1.36E+01	9.07E+00	1.63E+01	1.09E+01	9.67E+00	1.48E+01	8.77E+00	1.34E+01
F09	Mean	**9.15E+02**	9.17E+02	9.30E+02	9.94E+02	9.48E+02	9.27E+02	9.46E+02	9.30E+02	9.34E+02	9.42E+02	9.65E+02	9.48E+02
	Std	**5.82E+00**	8.30E+00	1.28E+01	8.23E+00	1.46E+01	1.01E+01	1.29E+01	1.28E+01	1.22E+01	1.65E+01	7.72E+00	1.66E+01
F10	Mean	**1.38E+03**	1.40E+03	1.62E+03	3.02E+03	1.97E+03	1.69E+03	2.30E+03	1.68E+03	1.66E+03	1.64E+03	2.46E+03	2.14E+03
	Std	**1.91E+02**	2.56E+02	2.50E+02	1.95E+02	3.30E+02	2.65E+02	3.80E+02	2.51E+02	2.44E+02	2.42E+02	2.59E+02	3.55E+02
F11	Mean	**1.64E+03**	1.69E+03	2.16E+03	3.52E+03	2.25E+03	1.88E+03	2.59E+03	1.93E+03	2.12E+03	2.22E+03	2.70E+03	2.26E+03
	Std	**2.35E+02**	4.17E+02	3.15E+02	2.38E+02	3.41E+02	3.02E+02	3.31E+02	3.25E+02	2.75E+02	3.27E+02	1.58E+02	3.46E+02
F13	Mean	**1.30E+03**	**1.30E+03**	**1.30E+03**	1.31E+03	**1.30E+03**	**1.30E+03**	**1.30E+03**	**1.30E+03**	**1.30E+03**	**1.30E+03**	**1.30E+03**	**1.30E+03**
	Std	**5.99E−02**	1.36E−01	1.50E−01	5.57E−01	1.07E+00	1.44E−01	1.19E+00	1.46E−01	1.24E−01	1.92E−01	7.37E−01	2.50E−01
F16	Mean	**1.60E+03**	**1.60E+03**	**1.60E+03**	**1.60E+03**	**1.60E+03**	**1.60E+03**	**1.60E+03**	**1.60E+03**	**1.60E+03**	**1.60E+03**	**1.60E+03**	**1.60E+03**
	Std	**1.30E−01**	5.44E−01	3.27E−01	3.24E−01	4.45E−01	5.19E−01	2.82E−01	3.69E−01	3.66E−01	3.31E−01	4.23E−01	4.82E−01
F19	Mean	**1.90E+03**	**1.90E+03**	**1.90E+03**	1.96E+03	1.91E+03	**1.90E+03**	1.91E+03	**1.90E+03**	**1.90E+03**	1.91E+03	1.92E+03	**1.90E+03**
	Std	**8.47E−01**	9.77E−01	9.91E−01	3.97E+01	2.06E+01	1.64E+00	9.13E+00	1.09E+00	8.95E−01	1.40E+00	1.12E+01	1.32E+00
F23	Mean	**2.50E+03**	2.63E+03	2.64E+03	**2.50E+03**	2.63E+03	2.63E+03	2.56E+03	2.64E+03	2.64E+03	2.62E+03	**2.50E+03**	2.63E+03
	Std	**0.00E+00**	3.86E+00	1.26E+01	**0.00E+00**	4.45E+01	6.66E−08	7.50E+01	7.02E+00	7.75E+00	4.29E+01	**0.00E+00**	7.00E+00
F24	Mean	**2.54E+03**	**2.54E+03**	**2.54E+03**	2.60E+03	2.58E+03	2.57E+03	2.58E+03	2.55E+03	**2.54E+03**	2.59E+03	2.60E+03	2.58E+03
	Std	2.65E+01	3.06E+01	1.39E+01	**0.00E+00**	2.45E+01	2.94E+01	2.07E+01	2.30E+01	1.94E+01	2.00E+01	5.95E+00	2.52E+01
F26	Mean	**2.70E+03**	**2.70E+03**	**2.70E+03**	**2.70E+03**	**2.70E+03**	**2.70E+03**	**2.70E+03**	**2.70E+03**	**2.70E+03**	**2.70E+03**	2.71E+03	2.71E+03
	Std	**4.27E−02**	4.63E−02	1.65E−01	2.19E+00	1.00E+00	1.82E+01	1.81E+01	1.82E+01	1.15E−01	2.20E−01	1.78E+01	3.45E+01
F27	Mean	**2.90E+03**	3.03E+03	3.09E+03	**2.90E+03**	3.18E+03	3.13E+03	3.13E+03	3.04E+03	3.12E+03	3.08E+03	3.29E+03	3.13E+03
	Std	**0.00E+00**	1.14E+02	7.78E+01	**0.00E+00**	1.14E+02	1.37E+02	1.53E+02	1.54E+02	9.22E+01	1.86E+02	9.00E+01	1.55E+02
F28	Mean	**3.00E+03**	3.27E+03	3.21E+03	**3.00E+03**	3.63E+03	3.60E+03	3.45E+03	3.27E+03	3.18E+03	3.40E+03	3.78E+03	3.61E+03
	Std	**0.00E+00**	8.93E+01	4.50E+01	**0.00E+00**	2.37E+02	2.70E+02	2.10E+02	7.57E+01	9.26E+00	1.37E+02	6.26E+02	1.85E+02
F29	Mean	**3.10E+03**	1.84E+05	1.19E+05	**3.10E+03**	1.05E+06	3.47E+03	3.13E+06	1.89E+05	4.83E+03	2.92E+05	1.41E+07	7.64E+05
	Std	**0.00E+00**	5.51E+05	4.37E+05	**0.00E+00**	2.09E+06	5.83E+02	4.89E+06	7.55E+05	2.23E+03	6.53E+05	1.94E+07	1.26E+06

In order to verify the statistical significance of the results of IGWO on the CEC2014 test function, Wilcoxon rank sum test is performed in this paper. In order to verify the performance of the proposed IGWO, a significance level of 5% is selected. The corresponding statistical results are presented in Table 11. In addition, Table 12 shows the average rankings generated by Friedman's test. The results show that the proposed IGWO significantly outperforms GWO, MFO, SHO, TSA, PSO, DOA, GJO, SOA, WOA, catGWO, and CPSOGSA. The overall analysis shows that the proposed IGWO is a better optimizer as compared to other methods.

**Table 11:** Wilcoxon rank sum test was performed on CEC2014 to obtain the statistical results.

Fun		GWO	MFO	SHO	TSA	PSO	DOA	GJO	SOA	WOA	catGWO	CPSOGSA
F03	*p*-value	7.48E−02	1.49E−04	3.02E−11	3.57E−06	2.13E−05	1.17E−02	4.83E−01	3.95E−01	3.02E−11	1.46E−10	9.52E−04
	Outcome	+	+	+	+	+	+	+	+	+	+	+
F06	*p*-value	5.69E−03	1.75E−05	3.02E−11	1.61E−10	2.15E−02	1.09E−10	6.53E−08	6.07E−11	3.69E−11	3.02E−11	3.83E−05
	Outcome	+	+	+	+	+	+	+	+	+	+	+
F08	*p*-value	7.51E−02	3.83E−05	3.02E−11	8.99E−11	1.56E−08	4.08E−11	1.31E−08	1.16E−07	1.07E−09	3.02E−11	8.10E−10
	Outcome	+	+	+	+	+	+	+	+	+	+	+
F09	*p*-value	4.92E−02	9.53E−07	3.02E−11	4.08E−11	5.86E−06	3.69E−11	3.81E−07	1.70E−08	2.61E−10	3.02E−11	4.97E−11
	Outcome	+	+	+	+	+	+	+	+	+	+	+
F10	*p*-value	8.30E−03	3.99E−04	3.02E−11	3.96E−08	4.94E−05	2.67E−09	4.64E−05	5.61E−05	4.64E−05	4.08E−11	2.67E−09
	Outcome	+	+	+	+	+	+	+	+	+	+	+
F11	*p*-value	8.42E−04	1.60E−07	3.02E−11	3.08E−08	2.62E−03	7.39E−11	9.52E−04	4.31E−08	2.60E−08	3.02E−11	2.60E−08
	Outcome	+	+	+	+	+	+	+	+	+	+	+
F13	*p*-value	7.73E−03	5.27E−05	3.02E−11	1.21E−10	5.79E−01	3.65E−08	8.15E−05	2.61E−10	1.01E−08	3.02E−11	4.69E−08
	Outcome	=	+	+	+	+	+	+	+	+	+	+
F16	*p*-value	2.64E−02	1.10E−08	8.10E−10	2.38E−07	2.42E−02	1.41E−09	1.86E−03	6.53E−08	1.56E−08	4.08E−11	1.25E−04
	Outcome	=	+	+	+	+	+	+	+	+	+	+
F19	*p*-value	3.11E−03	1.96E−01	3.02E−11	3.01E−07	6.63E−01	1.78E−10	1.95E−03	4.03E−03	3.16E−10	3.02E−11	7.70E−04
	Outcome	=	=	+	+	+	+	+	+	+	+	+
F23	*p*-value	1.21E−12	8.93E−13	NaN	1.21E−12	1.21E−12	1.46E−04	1.21E−12	1.21E−12	4.57E−12	NaN	5.24E−13
	Outcome	+	+	=	+	+	+	+	+	+	=	+
F24	*p*-value	8.53E−04	5.57E−03	1.33E−08	1.07E−07	1.43E−05	2.51E−07	1.68E−03	3.51E−02	1.20E−08	1.36E−07	4.80E−07
	Outcome	+	+	+	+	+	+	+	+	+	+	+
F26	*p*-value	3.48E-03	4.80E-07	3.02E-11	3.34E-11	1.71E-01	2.44E-09	4.18E-09	5.46E-09	3.32E-06	3.02E-11	1.75E-05
	Outcome	+	+	+	+	+	+	+	+	+	+	+
F27	*p*-value	1.33E−08	3.36E−11	NaN	3.36E−11	7.47E−10	4.13E−08	2.21E−06	3.36E−11	2.21E−06	1.21E−12	1.33E−08
	Outcome	+	+	=	+	+	+	+	+	+	+	+
F28	*p*-value	1.21E−12	1.21E−12	NaN	1.21E−12	3.36E−11	4.57E−12	1.21E−12	1.21E−12	1.21E−12	3.45E−07	1.21E−12
	Outcome	+	+	=	+	+	+	+	+	+	+	+
F29	*p*-value	1.21E−12	1.21E−12	NaN	1.21E−12	1.21E−12	1.21E−12	2.12E−13	2.12E−13	1.19E−12	1.21E−12	1.21E−12
	Outcome	+	+	=	+	+	+	+	+	+	+	+

**Table 12:** Experimental results of the Friedman test for CEC2014.

Algorithm	Single peak function	Multi-peak function	Combinatorial functions	Hybrid functions
IGWO	1.80	2.38	3.33	2.24
GWO	5.13	2.65	3.67	5.09
MFO	7.40	5.44	3.90	5.97
SHO	11.77	11.48	11.90	4.74
TSA	6.93	7.62	7.77	9.01
PSO	4.20	4.20	3.60	6.15
DOA	5.53	8.68	9.00	7.78
GJO	4.40	4.92	5.20	6.33
SOA	4.70	6.21	4.97	6.53
WOA	11.03	7.02	8.50	7.72
catGWO	8.67	10.71	10.60	8.99
CPSOGSA	6.43	6.67	5.57	7.44
*p*-value	2.52E−39	2.80E−35	2.57E−41	8.13E−30

## IGWO for engineering design problems

In order to highlight the performance and practical significance of the proposed IGWO algorithm, 2 classical engineering problems are used for further validation, i.e., automobile side impact design and three-bar truss design, and the results are compared with other algorithms. These 2 engineering problems are static single-objective constrained optimization problems. The example problem is generally expressed as (18).

$$\min F(x)$$

18 $$s.t.\left\{ {\begin{array}{*{20}c} {g_{i} (x) \le 0,i = 1,2,...,m} \\ {h_{j} (x) = 0,j = 1,2,...,n} \\ \end{array} } \right.$$

where, *F(x)* is the objective function ; *g*_*i*_*(x)* is an inequality constraint, and *h*_*j*_*(x)* is an equality constraint. In this paper, a penalty function is used to effectively handle the constraints, as expressed in (19).

19 $$\Phi (x) = F(x) \mp [\sum\nolimits_{i = 1}^{m} {l_{i} \cdot \max (0,g_{i} (x))^{\alpha } } + \sum\nolimits_{j = 1}^{n} {o_{j} \cdot |h_{j} (x)|^{\beta } ]}$$

where, $\Phi (x)$ denotes the final objective function, and *l*_*i*_ and *o*_*j*_ are two non-negative penalty coefficients. The larger the penalty coefficients, the better is the final optimization effect. The penalty coefficients in this paper are 1,000,000. *ɑ* and *β* are set to 2 and 1, respectively.

### Three-bar truss

**Figure 6:**
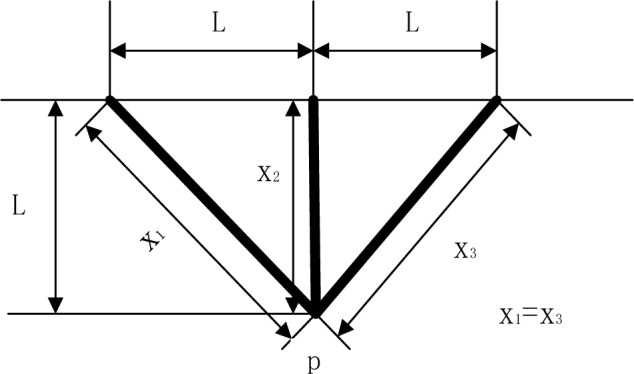
Schematic diagram of three-bar truss structure.

The three-bar truss design problem is one of the most classical design problems^8^. Its structure is shown in Fig. 6. The objective of the problem is to minimize the weight of the light rod structure. The constraints of the problem include stress, deflection, and buckling. Mathematically, the three-bar truss design problem is expressed in (20). The results obtained after parameter optimization of each algorithm are shown in Table 13.

20 $$\begin{gathered} \min f(x) = L(x_{2} + 2\sqrt 2 x_{1} ) \hfill \\ \begin{array}{*{20}c} {s.t.} & {g_{1} = \frac{{x_{2} }}{{2x_{1} x_{2} + \sqrt 2 x_{1}^{2} }}} \\ \end{array} P - \sigma \le 0 \hfill \\ \begin{array}{*{20}c} {} & {g_{2} = \frac{{x_{2} + \sqrt 2 x_{1} }}{{2x_{1} x_{2} + \sqrt 2 x_{1}^{2} }}} \\ \end{array} P - \sigma \le 0 \hfill \\ \begin{array}{*{20}c} {} & {g_{3} = \frac{1}{{x_{1} + \sqrt 2 x_{2} }}} \\ \end{array} P - \sigma \le 0 \hfill \\ \end{gathered}$$

where 0 ≤ *x*_*1*_*, x*_*2*_ ≤ 1, *L* = 100 cm, *P* = 2 KN/cm^2^, $\sigma$ = 2 KN/cm^2^.

**Table 13:** Comparison of results for the three-bar truss design problem.

Fun	X_1_	X_2_	Fitness
IGWO	0.79267752	0.39714963	**263.91802312**
GWO	0.80104225	0.3800476	264.5737234
CSO	0.81209756	0.35031812	264.727689
MFO	0.81632855	0.34990185	265.8827655
SCA	0.7631868	0.48754285	264.6161086
SOA	0.74583147	0.54570102	265.5230982
TSA	0.81510213	0.35051216	265.5969148
WOA	0.78051248	0.44259576	265.0218431
EGWO	0.8103815	0.35410616	264.6211177
Chaotic-GWO	0.809964	0.35139653	264.2320674
SCA-GWO	0.80534796	0.36897219	264.684022

Significant are in value [bold].

A comparison of the results obtained using the proposed IGWO with other swarm intelligence algorithms and the existing improved GWO algorithm is given in Table 13. The results show that the proposed IGWO is not worse than other algorithms in solving the three-bar truss design problem, and its optimal solution corresponds to the best fitness value of 263.91802312 [0.79267752, 0.39714963]. The results show that the algorithm is able to find the design solution with the lowest cost. Therefore, it is reasonable to conclude that the proposed algorithm is feasible in solving this type of problems.

### Automobile side impact design issues

The automobile side impact design problem is a classical single-objective minimization constrained design problem^9^. The design objective is to reduce the total weight of the car. The mathematical model of this problem is presented in (21).

$$\min f(x) = 1.98 + 4.9x_{1} + 6.67x_{2} + 6.98x_{3} + 4.01x_{4} + 1.78x_{5} + 2.73x_{7}$$

$$s.t.g_{1} = 1.16 - 0.3717x_{2} x_{4} - 0.00931x_{2} x_{10} - 0.484x_{3} x_{9} + 0.01343x_{6} x_{10} \le 1$$

$$\begin{gathered} g_{2} = 0.261 - 0.0159x_{1} x_{2} - 0.188x_{1} x_{8} - 0.019x_{2} x_{7} + 0.0144x_{3} x_{5} + 0.0008757x_{5} x_{10} \hfill \\ \begin{array}{*{20}c} {} & { + 0.080405x_{6} x_{9} + 0.00139x_{8} x_{11} + 0.00001575x_{10} x_{11} \le 0.32} \\ \end{array} \hfill \\ \end{gathered}$$

$$\begin{gathered} g_{3} = 0.214 + 0.00817x_{5} - 0.131x_{1} x_{8} - 0.0704x_{1} x_{9} + 0.03099x_{2} x_{6} - 0.018x_{2} x_{7} + 0.0208x_{3} x_{8} \hfill \\ \begin{array}{*{20}c} {} & { + 0.121x_{3} x_{9} - 0.00364x_{5} x_{6} + 0.0007715x_{5} x_{10} - 0.0005354x_{6} x_{10} + 0.00121x_{8} x_{11} \le 0.32} \\ \end{array} \hfill \\ \end{gathered}$$

$$g_{4} = 0.074 - 0.061x_{2} - 0.163x_{3} x_{8} + 0.001232x_{3} x_{10} - 0.166x_{7} x_{9} + 0.227x_{2}^{2} \le 0.32$$

$$g_{5} = 28.98 + 3.818x_{3} - 4.2x_{1} x_{2} + 0.0207x_{5} x_{10} + 6.63x_{6} x_{9} - 7.7x_{7} x_{8} + 0.32x_{9} x_{10} \le 32$$

21 $$g_{6} = 33.86 + 2.95x_{3} + 0.1792x_{10} - 5.05x_{1} x_{2} - 11x_{2} x_{8} - 0.0215x_{5} x_{10} - 9.98x_{7} x_{8} + 22x_{8} x_{9} \le 32$$

$$g_{7} = 46.36 - 9.9x_{2} - 12.9x_{1} x_{8} + 0.1107x_{3} x_{10} \le 32$$

$$g_{8} = 4.72 - 0.5x_{4} - 0.19x_{2} x_{3} - 0.0122x_{4} x_{10} + 0.009325x_{6} x_{10} + 0.000191x_{11}^{2} \le 4$$

$$g_{9} = 10.58 - 0.647x_{1} x_{2} - 1.95x_{2} x_{8} + 0.02054x_{3} x_{10} - 0.0198x_{4} x_{10} + 0.028x_{6} x_{10} \le 9.9$$

$$g_{10} = 16.45 - 0.489x_{3} x_{7} - 0.843x_{5} x_{6} + 0.0432x_{9} x_{10} - 0.0556x_{9} x_{11} + 0.000786_{11}^{2} \le 15.7$$

$$0.5 \le x_{1} \sim x_{7} \le 1.5;x_{8} ,x_{9} \in (0.192,0.345); - 30 \le x_{10} \sim x_{11} \le 30$$

As presented in Table 14, the proposed IGWO outperforms other algorithms and achieves the best results. It obtains the optimal solution corresponding to the optimal fitness value of 21.39473 [0.5, 0.88641, 0.5, 1.25781, 0.64809, 0.91372, 0.5, 1, 0.52483, 1.94790, 15.3719].

**Table 14:** Comparison of results for automobile side design problems.

Fun	X_1_	X_2_	X_3_	X_4_	X_5_	X_6_	X_7_	X_8_	X_9_	X_10_	X_11_	Fitness
IGWO	0.50000	0.88641	0.50000	1.25781	0.64809	0.91372	0.50000	1.00000	0.52483	1.94790	15.3719	**21.39473**
GWO	0.50029	1.10363	0.69363	1.28580	0.52101	1.19314	1.06101	0.74876	0.26735	0.10987	4.82565	25.61424
CSO	0.78544	0.56882	0.50000	1.34514	0.82107	0.86975	0.74586	0.89302	0.39281	0.89510	1.17997	22.00444
MFO	1.20667	0.53508	0.50569	1.26007	1.14441	0.92704	1.02223	0.80439	0.45074	6.59507	1.10872	24.87211
SCA	0.69494	0.85530	0.56237	1.35257	1.04657	1.24182	0.73188	0.79457	0.16779	7.12854	1.06108	24.30018
SOA	0.50000	0.94373	0.50000	1.24672	0.67642	0.78648	1.01788	0.88327	0.66043	0.89339	3.14055	23.19689
TSA	0.50315	0.89471	0.50000	1.40533	0.86396	0.84245	0.59580	0.86533	0.06579	0.12364	2.06338	22.70296
WOA	0.66573	0.69895	0.50000	1.50000	1.19847	0.99347	0.56123	1.00000	0.0000	8.71911	4.37216	23.07446
EGWO	0.55338	1.10957	0.57088	1.35356	0.79095	0.96991	0.86405	0.71495	0.23680	2.38632	9.70454	25.27173
Chaotic-GWO	0.84252	0.50000	0.50000	1.36186	0.83852	0.86445	0.57679	0.94066	0.24622	4.55062	12.30842	21.46164
SCA-GWO	0.52564	0.79622	0.50000	1.50000	1.50000	1.50000	0.50000	1.00000	0.55779	1.21649	0.25375	23.40641

In summary, the results show that the proposed IGWO outperforms other compared algorithms. It can be seen that IGWO not only improves the global search capability, but also helps to prevent the search from falling into local minima and effectively solves engineering design problems.

## Conclusions

In this paper, a multi-strategy fusion improved gray wolf algorithm IGWO is proposed by combining three different strategies, including optimizing the initial population, modifying the nonlinear control parameters, and the search mechanism. These modifications effectively balance the exploration and exploitation, while maintaining a high convergence rate. In order to verify the effectiveness of the improved algorithm, the proposed IGWO is assessed using 23 benchmark test functions and 15 CEC test functions of 4 different complexity levels. The corresponding test results are statistically analyzed. The results show that the improved algorithm performs well and enhances the convergence speed and search accuracy. In order to verify the engineering applicability of the improved algorithm, in this paper, the improved algorithm is applied to the automobile side design and the three-bar truss design problems. The results show that the proposed method outperforms other swarm intelligence optimization algorithms in solving the engineering optimization problems.

## Data Availability

All data generated or analysed during this study are included in this published article.
